# Buffalo milk and rumen fluid metabolome are significantly affected by green feed

**DOI:** 10.1038/s41598-022-25491-w

**Published:** 2023-01-25

**Authors:** G. Neglia, A. Cotticelli, A. Vassetti, R. Matera, A. Staropoli, F. Vinale, A. Salzano, G. Campanile

**Affiliations:** 1 Department of Veterinary Medicine and Animal Production, University of Naples “Federico II”, 80137 Naples, Italy; 2 Institute for Sustainable Plant Protection, National Research Council, 80055 Portici, NA Italy; 3 Department of Agricultural Sciences, University of Naples “Federico II”, 80055 Portici, NA Italy

**Keywords:** Metabolomics, Animal behaviour, Animal physiology

## Abstract

The use of green feed for livestock breeding is an important strategy to encounter both the increasing demand for animal derived products and the perceptions of the consumers regarding animal welfare and sustainability. The aim of this study was to compare different feeding strategies in lactating water buffaloes by using a metabolomic approach. The study was carried out on 32 milking buffaloes that were randomly divided into two groups for a total period of 90 days (3 sampling times). DD Group (dry diet) received a standard total mixed ratio (TMR) characterized by dry forages and concentrates; ZG Group (zero grazing) fed an isoenergetic and isoproteic diet obtained using 30% of sorghum as green forage. Samples of milk and rumen fluid were analyzed by liquid chromatography—mass spectrometry (LC–MS) techniques. Data analyses revealed the presence of several differentially accumulated metabolites and among these, ten compounds were putatively identified in milk samples (i.e. l-carnitine, acetylcarnitine, propionylcarnitine, butyrylcarnitine, 2-methylbutyroylcarnitine, 2-hexenoylcarnitine, hexanoylcarnitine, glycerophosphocholine, δ-valerobetaine and γ-butyrobetaine) and four in rumen fluid (3-(2-hydroxyphenyl) propanoate, Indole-3-acrylic acid, oleamide (cis-9,10-octadecenoamide) and 20-carboxy-leukotriene B4). The modulation of these molecules in buffalo milk is significantly related to the green/dry based feeding and some the natural compound detected could be considered as health-promoting nutrients.

## Introduction

In the last years an increased consumer’s sensibility has been noted about the methods of production of animal derived foods^1^. Particularly, several concerns have arisen towards livestock intensive breeding conditions, which are considered one of the causes of environmental pollution as well as disrespectful of animal welfare, although a direct relationship among the intensification of livestock and these topics has not been provided^2,3^. Whatever may or may not be the truth of these speculations, the challenge of future techniques is to focus on the sustainability of livestock systems, together with high quality derived products and animal welfare^4^. Furthermore, in order to encounter the growing global demand for animal derived food, more land (from 0.2 to 1 billion additional hectares) is required until 2050^5^.

The use of grassland for livestock breeding could be an appropriate strategy to increase the competitiveness of the farms, since there is a huge amount of land, named “marginal areas”, where arable production cannot be carried out and grazing may represent a tool to increase its utilization and efficiency^6,7^. However, excluding marginal areas, when lands are exploited for grazing, low efficiency and production are recorded, leading to low economic and environmental sustainability of extensive breeding conditions^7^.

An alternative and valid strategy is the “*zero grazing*” technique, recently developed to increase livestock sustainability by using grassland. Also known as “*cut and carry*”, it consists in grass harvesting and administration to housed animals as total mixed ration (TMR), in a group pen situation^8,9^. This appealing technique allows to both reduce feeding costs and to introduce high quality forage in the diet of the animals, since grass is a valuable source of mineral and vitamins, with advantages on milk quality^10^. In fact, recent studies, carried out on dairy buffaloes, demonstrated that green forage increased the concentration of some health-promoting biomolecules in milk^11^.

Milk is a complex biological fluid, consisting of water and several nutrients, such as lipids, carbohydrates, proteins, vitamins, minerals, and some small secondary metabolites^12^. Furthermore, milk chemical composition depends on many intrinsic and external factors that can influence its characteristics^13^. Some studies focused on the characterization of the chemical composition of milk, evaluating the effect of different TMR and types of grazing on milk parameters^14,15^. In particular, some authors investigated the relationship between forages and milk metabolite composition to identify discriminating factors in cow's milk through a metabolomic approach^12^.

Bovine rumen activity and the symbiotic relationship between the host and the microbiota have a direct effect on physiological parameters of animals and milk composition^16,17^. Rumen activity depends on a complex anaerobic environment composed by a variety of microorganisms, mainly bacteria, which convert feed material into energy^17^. Jami et al.^18^ explored rumen composition of dairy cows finding a connection between bacterial components of the rumen microbiota and the physiological parameters of the animal host during lactation.

Metabolomics is the systematic study of the chemical profile of a biological system, and is composed by all the small molecules, intermediates and products of a metabolism^19^. The metabolome is closely bound to the genotype of an organism, its physiology and its environment. For this reason, metabolomics offers the opportunity to study genotype–phenotype and genotype-environment relationships^20^. In this regard, analytical techniques such as liquid chromatography—mass spectrometry (LC–MS and LC–MS/MS), nuclear magnetic resonance (NMR) spectroscopy and gas chromatography-mass spectrometry (GC–MS), together with bioinformatics tools, have been successfully applied in livestock researches to screen the metabolomic profile of complex matrices, such as milk and other biofluids, to discover factors or biomarkers of feed efficiency and residual feed intake, to study dietary responses to different feeds, milk quality and safety and fertility^12,21–27^. In a recent study a metabolomic approach was also used to characterize buffalo milk and its derived mozzarella cheese, produced in protected denomination of origin (PDO) areas vs. non-PDO counterparts^28^. It cannot be ruled out that the use of in loco produced forages may allow to further characterize the milk in terms of traceability, and this is particularly important for PDO products such as “Mozzarella di Bufala Campana” cheese. Also, metabolomic studies on ruminal fluid may allow the evaluation of the impact of specific diets on buffaloes.

Therefore, the present work aims to evaluate the effect of different feeding system on buffalo milk using a metabolomic approach and to assess the impact of feeding on metabolome rumen fluid.

## Results

### Dry matter intake

Total dry matter intake (DMI) was similar between DD and ZG buffaloes (16.64 ± 0.22 and 16.73 ± 0.28 kg/day respectively for DD and ZG group, p = 0.81). Similarly, body condition score (BCS) did not differ (*p* > 0.05) between ZG and DD buffaloes (7.62 ± 0.03 vs. 7.51 ± 0.05 respectively).

### Milk yield and quality

As showed in Table 1, average milk yield and energy corrected milk (ECM) throughout the experimental period did not differ significantly between the two groups (12.43 ± 0.09 vs 13.05 ± 0.09 kg respectively for DD and ZG group).

**Table 1:** Average milk yield and milk quality traits in buffaloes of zero grazing (ZG) and dry diet (DD) Group.

Group	Milk yield (kg)	Fat content (%)	Protein content (%)	Energy corrected milk (kg)	Cheese yield* (%)	Somatic cell count (n)
DD	12.43 ± 0.09^a^	8.83 ± 0.31^a^	4.32 ± 0.78^a^	21.12 ± 0.92^a^	25.10 ± 0.50^a^	196.84 ± 59.79^a^
ZG	13.05 ± 0.09^a^	8.16 ± 0.30^a^	4.22 ± 0.10^a^	21.01 ± 0.96^a^	23.93 ± 0.58^a^	286.04 ± 76.54^a^

Data regarding energy corrected milk (ECM), cheese yield and somatic cell count were also reported.

Data are reported as mean ± Standard error.

*Cheese yield = [fat (g/kg) × 1.23) + (protein (g/kg) × 3.5)−0.88]^29^. Values with different superscripts in the same column are significantly different (^a,b^, *p* < 0.01).

No differences were found on milk quality traits, neither on average total production, nor on monthly basis.

### Milk metabolome

**Figure 1:**
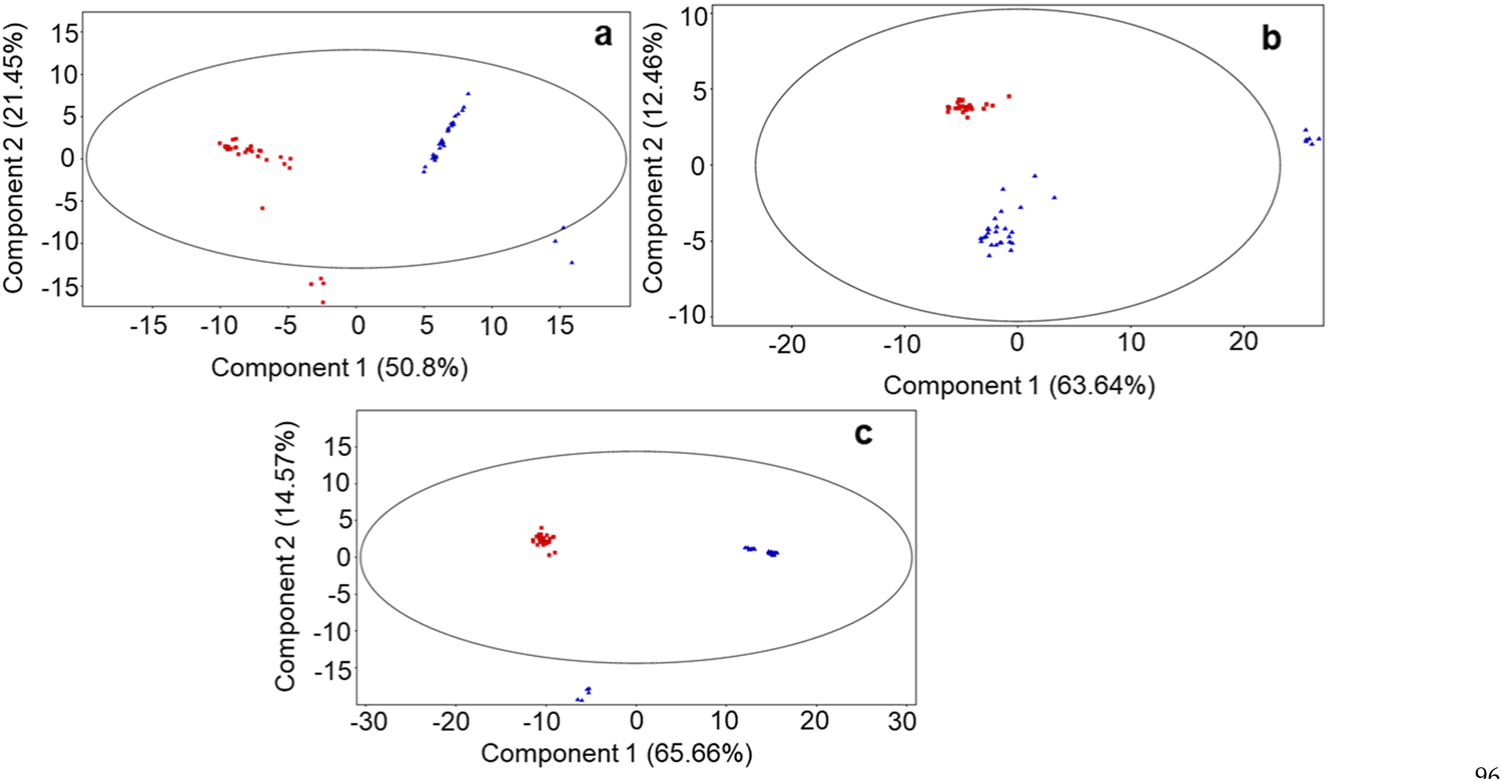
PCA score plots of the LC–MS data acquired for the three milk samplings from buffaloes DD Group fed a total mixed ratio (TMR) (red squares) and ZG Group fed TMR + 30% of green forage (blue triangles). (**A**) (June sampling): PC1 accounts for 50.85% and PC2 21.45% of total variance; (**B**) (July sampling): PC1 variance 63.64% and PC2 variance 12.46%; C) (August sampling): PC1 variance 65.66% and PC2 variance 14.57%.

The study of milk metabolomic profile revealed a compositional differences between DD and ZG buffalo groups. As resulted from the multi- and univariate analysis of the LC–MS dataset, DD and ZG groups cluster separately from each other when comparing samples collected at the same time point (Fig. 1), differing in the abundance of specific metabolites (Table 2). Moreover, each multivariate analysis showed an unsupervised separation within the two groups, particularly evident in the first sampling time (June, Fig. 1a); the presence of outliers (Fig. 1) is related to the absence of several unidentified metabolites and amino acid derivatives. The trend is due to individuals’ unavoidable variability since the single animal, the personnel interacting with the animals, the animals and/or environmental microbiome, and several other factors resist standardization^30^.

**Table 2:** Metabolites obtained from LC–MS data (positive mode), that are differentially accumulated in milk samples from buffaloes of DD Group (fed a total mixed ratio (TMR)) and ZG Group (fed TMR + 30% of green forage) analyzed for 3 months (June, July and August).

Compound	Monoisotopic mass (Da)	Regulation DD vs ZG					
		June	July	August	LnFC (June)	LnFC (July)	LnFC (August)
γ-Butyrobetaine	145.1098	Down	Down	Down	− 18.1088	− 7.8222	− 16.2603
δ-Valerobetaine	159.1254	n.d.	Down	Down	n.d.	− 2.98936	− 14.3323
l-Carnitine	161.1048	n.d.	Down	Down	n.d.	− 3.20882	− 14.6969
Acetylcarnitine	203.1163	Up	Down	Down	21.28232	− 2.96594	− 14.6381
Propionylcarnitine	217.1312	n.d.	Down	Down	n.d.	− 14.4192	− 10.7945
Butyrylcarnitine	231.1485	Up	Down	Up	22.13542	− 2.93003	7.460587
2-Methylbutyrylcarnitine	245.1644	Up	Down	Up	19.65461	− 3.0367	5.092634
Glycerophosphocholine	257.1036	Down	Down	n.d.	− 12.3487	− 3.10791	n.d.
2-Hexenoylcarnitine	257.1664	Up	n.d.	n.d.	15.89597	n.d.	n.d.
Hexanoylcarnitine	259.1796	Down	n.d.	n.d.	− 15.6068	n.d.	n.d.

Identifications were performed by comparing results with known compounds present in freely available electronic databases.

Milk Composition Database (MCDB) and Bovine Metabolome Database (BMDB) were used for the identification. Up, up regulated; Down , down regulated; n.d. , compounds not detected after statistical analysis. LnFC, Natural logarithm of Fold Change.

Among all the compounds differentially detected (118, 127 and 215 compounds detected in June, July, and August sampling respectively, Supplementary tables S1–S3), ten have been putatively identified and reported in Table 2. These metabolites belong to the class of compounds known as carnitine and derivatives (i.e. acetylcarnitine or butyrylcarnitine), coline derivates (glycerophosphocholine) and carnitine precursor (γ-butyrobetaine). It is possible to observe that the metabolites derived from carnitine are more abundant in milk samples from the group of buffaloes fed ZG. The same trend can be noticed for glycerophosphocholine.

**Figure 2:**
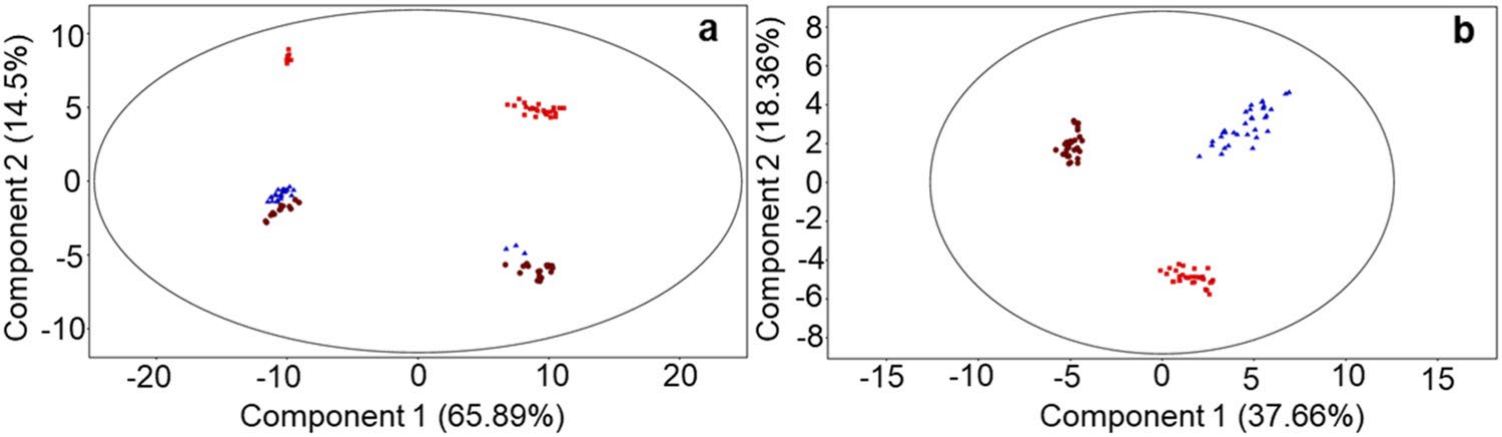
PCA score plots of the LC–MS data acquired for milk samples collected in June (brown circles), July (blue triangles) and August (red squares) from buffaloes DD Group fed a total mixed ratio (TMR) and ZG Group fed TMR + 30% of green forage. (**A**) DD Group: PC1 accounts for 65.89% and PC2 14.5% of total variance; (**B**) ZG Group: PC1 variance 37.66% and PC2 variance 18.36%.

Moreover, a longitudinal analysis was performed to investigate differences during the experimental period (from June to August) within each group of buffaloes, DD and ZG. The results of multivariate analyses (PCA) are reported in Fig. 2. Milk samples collected from buffaloes belonging to the ZG Group showed a separation of August group (red) from June and July groups (brown and blue respectively) which are closer in the components space (Fig. 2b), whereas analysis of DD Group revealed a clustering of June and July samples (brown and blue, respectively) clearly separated along PC2 from August (red, Fig. 2a). The same trend is also noticeable along component 1 for the two sub-groups resulting from the biological variation of the samples^30^.

Univariate analyses of metabolic profiles for the three sampling times resulted in the detection of 145 and 66 differentially accumulated compounds in DD and ZG Group respectively (Supplementary Tables S4, S5), among which 7 were putatively identified for DD and 4 for ZG. These compounds, reported in Tables 3 and 4, belong to the class of carnitine and derivatives and choline derivatives. It is possible to notice that all molecules identified in DD Group, with the exception of glycerophosphocholine, are less abundant in August compared to June, whereas the same trend does not apply to ZG Group.

**Table 3:** Metabolites obtained from LC–MS data (positive mode), that are differentially accumulated in milk samples from buffaloes of DD Group (fed a total mixed ratio (TMR)) for the whole experimental period (June, July and August).

Compound	Monoisotopic mass (Da)	Regulation			
		July vs June	August vs June	LnFC (July vs June)	LnFC (August vs June)
δ-Valerobetaine	159.1254	Up	Down	7.1799765	− 3.2817602
l-Carnitine	161.1048	Up	Down	7.0949345	− 3.4304905
Acetylcarnitine	203.1163	Down	Down	− 14.375848	− 25.062004
Propionylcarnitine	217.1312	Up	Down	9.049169	− 3.0859761
Butyrylcarnitine	231.1485	Up	Down	6.968273	− 3.7872849
2-Methylbutyrylcarnitine	245.1644	Up	Down	8.384206	− 3.6744347
Glycerophosphocholine	257.1036	Up	Up	28.734497	10.195721

Identifications were performed by comparing results with known compounds present in freely available electronic databases.

Milk Composition Database (MCDB) and Bovine Metabolome Database (BMDB) were used for the identification. Up, up regulated; Down, down regulated. LnFC, Natural logarithm of Fold Change.

**Table 4:** Metabolites obtained from LC–MS data (positive mode), that are differentially accumulated in milk samples from buffaloes of ZG Group (fed TMR + 30% of green forage) for the whole experimental period (June, July and August).

Compound	Monoisotopic mass (Da)	Regulation			
		July vs June	August vs June	LnFC (July vs June)	LnFC (August vs June)
γ-Butyrobetaine	145.1099	Up	Up	3.0676727	3.0186539
Propionylcarnitine	217.1312	Up	Down	1.3566017	− 16.55081
Butyrylcarnitine	231.1485	Up	Up	23.485516	1.4046552
Glycerophosphocholine	257.1036	Up	Down	10.945495	− 10.318822

Identifications were performed by comparing results with known compounds present in freely available electronic databases.

Milk Composition Database (MCDB) and Bovine Metabolome Database (BMDB)were used for the identification. Up, up regulated; Down , down regulated. LnFC , Natural logarithm of Fold Change.

**Figure 3:**
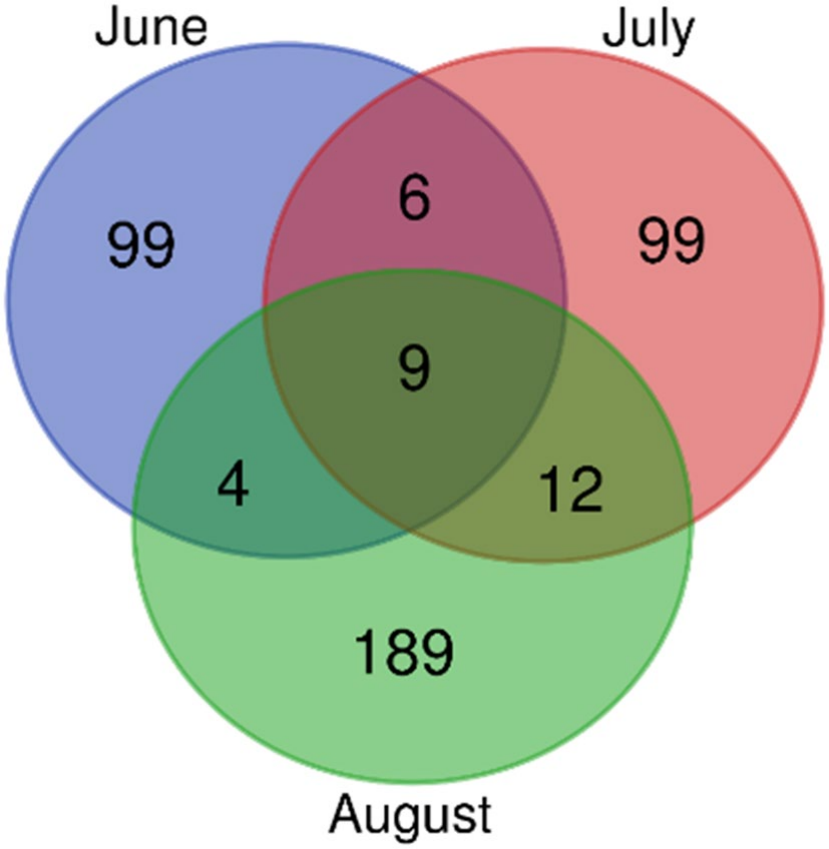
Venn diagram of differentially accumulated metabolites for each sampling time (June, July, August) of buffaloes fed TMR and TMR + 30% green forage.

In order to highlight differences among sampling times in terms of common molecules, a Venn diagram (Fig. 3) was generated (Bioinformatics & Evolutionary Genomics**,** http://bioinformatics.psb.ugent.be/webtools/Venn/).

As reported in Fig. 3, 9 compounds are common to all sampling times, while 99, 99 and 189 molecules were exclusively detected in June, July and August respectively. Among these common metabolites, it was possible to identify four metabolites: acetylcarnitine, butyrylcarnitine, 2-methylbutyrylcarnitine and γ-butyrobetaine (Table 2).

### Rumen fluid metabolome

**Figure 4:**
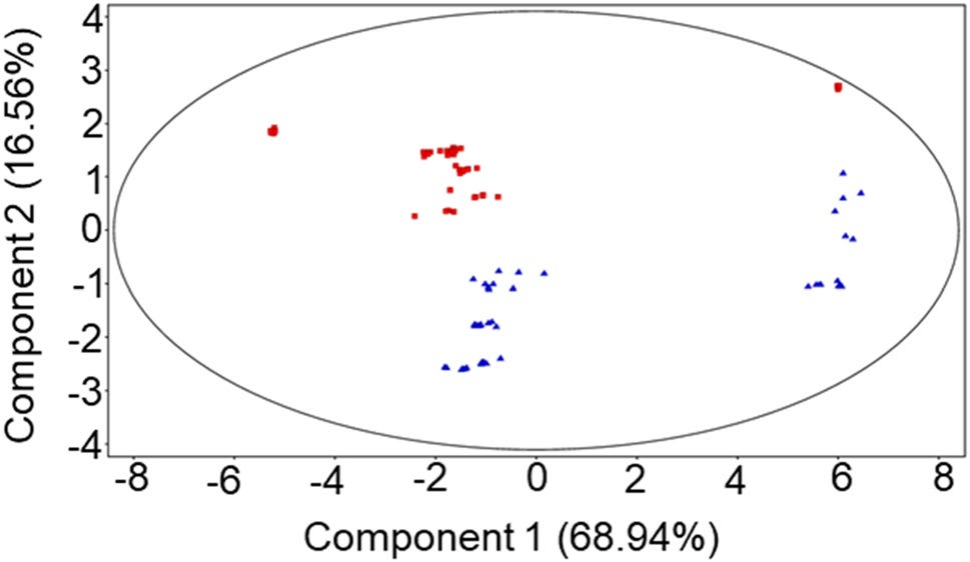
PCA score plots of the LC–MS data acquired for rumen fluid extract obtained from DD Group fed a total mixed ratio (TMR) (red squares) and ZG Group fed TMR + 30% of green forage (blue triangles). PC1 accounts for 68.94% and PC2 16.56% of total variance.

Multivariate analysis was carried out to detect intrinsic clustering between rumen fluid samples. PCA scores plot reported in Fig. 4 revealed a separation of the two groups (DD in red and ZG in blue) along PC2, which accounts for 16.56% of the total variance. However, a second trend was evident along PC1 (68.94% of total variance), suggesting a separation within each group of samples. DD rumen fluid samples formed a second cluster related to the absence of one unidentified metabolite (molecular formula C_18_H_12_N_2_O_2_). ZG samples spread along PC1, and this trend is due to an enhanced biological variability^30^. Particularly, a subgroup of samples, all belonging to one individual, showed a different metabolic profile, in which most of the differential compounds were absent (e.g., 3-(2-hydroxyphenyl)-propanoate, 20-Carboxy-leukotriene B4 and other 8 unidentified molecules).

Among the molecules differentially accumulated (16, Supplementary Table S6), four have been putatively identified: 3-(2-hydroxyphenyl)-propanoate, indole-3-acrylic acid, oleamide (cis-9,10-octadecenoamide) and 20-carboxy-leukotriene B4 (Table 5). All these compounds are down regulated in dry *vs* green feeding.

**Table 5:** Metabolites obtained from LC–MS data (positive mode), that are differentially accumulated in ruminal fluid samples from buffaloes of DD Group (fed a total mixed ratio (TMR)) and ZG Group (fed TMR + 30% of green forage).

Compound	Monoisotopic mass (Da)	Regulation DD vs ZG	LnFC (DD vs. ZG)
3-(2-hydroxyphenyl)-propanoate	165.0338	Down	− 19.4548
Indole-3-acrylic acid	187.0635	Down	− 4.18757
Oleamide	281.2724	Down	− 3.20791
20-Carboxy-leukotriene B4	366.2024	Down	− 6.38134

Identifications were performed by comparing results with known compounds present in a freely available electronic database.

Bovine Metabolome Database (BMDB) was used for the identification. Up , up regulated; Down, down regulated. LnFC, Natural logarithm of Fold Change.

## Discussion

Several concerns have been recently expressed regarding the environmental and economical sustainability of livestock^2^. This is mainly due to the intensification of livestock systems, that caused the concentration of animals in restricted areas, leading to both poor utilization of pasture and lower animal welfare^3,4^. The application of *cut and carry* (or *zero grazing*) technique represents an interesting and innovative method to increase both economic and environmental sustainability of the farm^8^. In the present study milk yield recorded in ZG Group was similar to that recorded in DD Group. In a recent experiment carried out in dairy cattle, the animals were divided into three groups: grazing, housed fed zero grazing and housed fed grass silage. In this case a dry diet has not been considered; the authors recorded a higher DMI and milk yield in grazing cows compared to their “*cut and carry*” counterparts^10^. Contrarily, a drop in milk yield in grazing cows was observed by other authors, due to the lower DMI at pasture^31^. Similarly to our results, in a previous trial carried out on dairy buffaloes no differences were recorded when alfalfa green forage was administered in comparison to dry diet^11^. Furthermore, in the present study no differences were recorded in terms of dry matter intake between DD and ZG groups, as previously observed in both dairy buffaloes and dairy cattle^11,32^. A similar BCS has been also recorded, suggesting a better efficiency in diet assimilation and utilization in the former.

In the present work, no differences were recorded both for fat and protein content of milk from zero grazing buffaloes with respect to the ones receiving dry diet. A clear scenario has not been described concerning the influence of fresh forage on milk quality traits: some authors recorded a higher fat content in milk produced under intensive/semi-intensive system, whereas other authors disagree and report no differences^32–35^. A clear effect of green forage has been described on milk fat characteristics in both cattle and buffalo, since green forage is a natural source of vitamins, C18:3n-3 and other unsaturated fatty acids^36,37^. In particular, the inclusion of 20 kg (26.5% of DM) of sorghum as green forage in the diet of lactating buffaloes increased unsaturated fatty acid concentration and simultaneously decreased that of saturated fatty acid. Furthermore, these characteristics were also recorded in derived mozzarella cheese, and did not influence consumer’s acceptance^37^.

The main aim of this trial was to get advantage of a metabolomic approach to evaluate the influence of different feeding strategies on milk metabolites, by comparison of the two groups within each time point and of the three samplings withing each feeding group. Results showed strong differences between the two sets of milk samples. For each sampling time, i.e. June, July and August, several statistically different metabolites were found: 118, 127 and 215 respectively. Whereas, for each feeding group, i.e. DD and ZG, respectively 145 and 66 differential metabolites have been detected. Moreover, nine compounds were common to all sampling times, while 99, 99 and 189 were exclusively detected in June, July and August respectively. Among the differently accumulated natural compounds, several were identified by comparison with online databases and literature and classified as belonging to carnitine and derivatives group.

Carnitine, its derivates (l-carnitine, acetylcarnitine, propionylcarnitine, butyrylcarnitine, 2-methylbutyrylcarnitine, hexanoylcarnitine and 2-hexenoylcarnitine) and precursor (γ-butyrobetaine) differ, during all the months object of this study, in the animals fed a total mixed ratio (TMR) (DD Group) and in animals fed with the same diet of DD Group but with the inclusion of the 30% of alfalfa green forage (ZG Group). In particular, an upregulation trend of these compounds has been noticed in milk collected from animals subjected to green forage integration, suggesting that the feeding strategy has an impact on the abundance of bioactive compounds as also reported by Bellassi et al.^38^.

Δ-valerobetaine (DVB) belongs to the class of organic compounds known as straight chain fatty acids and it was firstly detected in ruminant milk and meat by Servillo et al.^39^ They demonstrated that this compound is obtained in rumen by transformation of trimethyllysine (TML) which is a constituent of animal feedstuff from vegetal origin. TML is particularly abundant in alfalfa green forage^40,41^. The DVB was detected in two sampling times such as July and August and resulted more abundant in milk samples belonging to ZG Group. The addition of green forage could enrich milk due to a higher concentration of TML, thus leading to a higher amount of DVB.

Γ-butyrobetaine (GBB), also known as 3-dehydroxycarnitine, is a precursor of l-carnitine derivate from γ-aminobutyric acid (GABA)^42^. It is a substrate of γ-butyrobetaine hydroxylase/dioxygenase (also known as BBOX) which catalyzes the formation of l-carnitine from γ-butyrobetaine, the last step in the l-carnitine biosynthesis pathway. The GBB was detected in all samples and at all times, as down-regulated in samples belonging to DD Group. This molecule is an important source for being the precursor of carnitine, and higher levels of GBB may have an impact on its production leading to a higher concentration of the final product^40^.

l-Carnitine is a water-soluble quaternary amine found in plant, animal, and microbial kingdoms, that participates in several enzymatic reactions. It plays an important role in the intermediary metabolism of fatty acids and in the beta-oxidation of long-chain fatty acids in the mitochondria; it regulates CoA concentration and removal of the produced acyl groups. Transformation of acyl-CoA into acylcarnitine is an important system also for removing the toxic acyl groups^43,44^. l-Carnitine can be converted into l-acetylcarnitine through its interaction with the enzyme carnitine *O*-acetyltransferase. Our findings showed that l-carnitine and l-acetylcarnitine are more abundant in milk produced by animals fed the inclusion of the 30% of alfalfa green forage (ZG Group) at second and third sampling time. l-carnitine was not detected at first sampling time, while l-acetylcarnitine was more abundant in DD Group. This result could be explained by considering the conversion of carnitine into acetylcarnitine.

In cattle, propionylcarnitine is involved in the metabolic pathway called the oxidation of branched-chain fatty acids pathway^45^. Propionylcarnitine is always down-regulated in samples belonging to DD Group, except for June in which it was not detected.

Butyrylcarnitine is part the class of organic compounds known as acylcarnitines^40^. These are compounds containing a fatty acid in which the carboxylic acid is linked to the carnitine through an ester bond. Thus, butyrylcarnitine is considered to be a fatty ester lipid molecule. Butyrylcarnitine is always up-regulated in milk from animals fed a total mixed ratio (DD Group), except for July (second sampling time).

Hexanoylcarnitine and 2-hexenoylcarnitine are acylcarnitines. 2-hexenoylcarnitine was only detected in June and was more abundant in DD Group compared to ZG Group. Hexanoylcarnitine, as well as 2-hexenoylcarnitine, was found only in June samples and was more abundant in milk produced by animals belonging to ZG Group. It could be possible that hexanoylcarnitine was converted to 2-hexenoylcarnitine and then degraded to other products.

2-Methylbutyroylcarnitine is an acylcarnitine. Specifically, it is a 2-methylbutanoic acid ester of carnitine^39^. In this study 2-methylbutyroylcarnitine resulted in general more abundant in animals of DD Group except for the second sampling (July). Servillo et al.^39^ detected this molecule in cow and buffalo milk, together with several other carnitine derivates such as acetylcarnitine, propionylcarnitine, butyrylcarnitine, isobutyrylcarnitine and 3-methylbutyrylcarnitine (isovalerylcarnitine), which resulted with higher level in buffalo milk than cow milk. The effect of green feed was examined in in milk of dairy buffaloes in a study of Salzano et al.^11^. Buffaloes fed with TMR + alfalfa green feed (30% of diet) had a higher level of l-carnitine, acetyl-l-carnitine, propionyl-l-carnitine and δ-valerobetaine in milk.

Glycerophosphocholine represents the main form of choline storage in the cytosol together with phosphocholine. This derivative of choline is an essential organic osmolyte in renal medullary cells, is involved in osmoadaptation and in the reduction of urea toxicity on bioactive enzymes and other macromolecules^46,47^. The intracellular accumulation of osmolytes is due to: (1) an increased uptake of myoinositol and betaine; (2) the biosynthesis of sorbitol and glycerophosphocholine; (3) a reduced degradation of glycerophosphocholine; (4) and a reduced osmolyte release. Phosphatidylcholine breakdown, present in various tissues, including mammary tissue, produces glycerophosphocholine as major metabolite^48^. This molecule was identified in first two sampling times as down-regulated for DD Group compared to ZG Group. High milk GPC values are connected with a low ketosis incidence in cows of different breeds (Holstein–Friesian, Brown Swiss, and Simmental Fleckvieh) and for animals in different lactations, with observed odds ratios between 1.5 and 2.38^46^. It is possible that GPC and phosphocoline could became prognostic biomarker for risk of ketosis^47^.

With regards to the longitudinal analysis of the two groups along three time points, identified metabolites were seven for DD Group (carnitine, its acetyl, proprionyl, butyryl and 2-methylbutyryl derivatives, GPC, and DVB) and four for ZG Group (GBB, GPC, butyrylcarnitine and proprionylcarnitine). A clear trend is demonstrated in milk samples from buffaloes of the dry group as the metabolites are more abundant in July compared to June (with the exception of acetylcarnitine which is down-regulated) and less abundant in August compared to June (except for GPC that is upregulated). Total mixed ratio may have affected milk metabolome by inducing a major production of these compounds during the first months of feeding that were subsequently converted into other products useful for the animal, thus justifying the lower abundance found in August vs June comparison. Although most of carnitines level did not statistically differ along the experimental period, a similar trend can be noticed in ZG group. In fact, all the detected molecules are more abundant in July compared to June, whereas a higher variability is present in August vs June comparison. Nonetheless there may be a correlation between the diet and the period during which the diet is given.

γ-Butyrobetaine shows a clear trend connected to the diet (Table 2) and among the unidentified molecules there are four that resulted common to each sampling time, together with acetylcarnitine, butyrylcarnitine, 2-methylbutyroylcarnitine and γ-butyrobetaine. Further studies are needed to purify (based on extensive application of chromatographic methods) and identify these metabolites, but they may be considered as biomarkers that could confirm milk origin and quality as buffalo milk and not cows.

Rumen fluid metabolites, identified and down regulated on DD vs ZG samples, for which a positive effect has been registered using fresh feed, belong to amino acid (3-(2-hydroxyphenyl) propanoate and indole-3-acrylic acid) and fatty acid (oleamide and 20-Carboxy-leukotriene B4) metabolism. In particular, 3-(2-hydroxyphenyl) propanoate, also known as melilotic acid or melilotate, is produced by the anaerobic bacterium *Clostridium xylanolyticum* from cinnamic acid^17^.

Indole-3-acrylic acid is a product of tryptophan metabolism that improve intestinal epithelial barrier function and alleviates inflammatory responses^49^. The gut microbiota can directly utilize tryptophan, which is metabolized into indole, tryptamine, indole acid derivatives, skatole and indicans^50^.

Oleamide (cis-9,10-octadecenoamide) is a primary amide derived from fatty acids. From oleoyl-CoA and glycine, *N*-oleoylglycine is obtained through an enzyme not yet identified which is modified to oleamide by PAM enzyme^51^. It has been identified in rumen fluid by Artegoitia et al.^52^ as a potential biomarker for cattle feed efficiency.

20-Carboxy-leukotriene B4 is a long-chain fatty acid identified also in rumen fluid by Artegoitia et al.^52^ as a potential biomarker for cattle feed efficiency, and an omega-oxidized metabolite of leukotriene B4 (LTB4). Leukotrienes are metabolites of arachidonic acid and derived from the 5-lipoxygenase (ALOX-5) activity in the lipoxygenase pathway.

In this investigation, the use of green feed for diet of dairy buffaloes affected the chemical profile of both milk and rumen fluid samples, resulting in a modulation of several metabolites, mainly belonging to amino acid and fatty acid derivative classes. These natural compounds have been detected in milk and, some of them, as related compounds in rumen fluid. This examination of rumen fluid and milk metabolome suggests that a ‘omics approach is a valid method to study the changes in animal’s metabolism and in particular to find specific correlations between the physiological parameters of buffaloes, such as milk composition, and the products of rumen fermentation. Further studies are needed to evaluate this causative relationship and characterize some unknown molecules not identified with the LC–MS based metabolomics and that can be considered as biomarkers for the use of green feed in livestock farming.

## Materials and methods

### Farm, animals and feeding regimen

All the procedures were performed according to standard veterinary practices and received formal approval from the Ethical Animal Care and Use Committee of the University of Naples Federico II, Italy (Protocol Number. 996072017). The trial was carried out in a commercial buffalo farm located in the South of Italy, where about 800 animals were bred for a total period of 90 days from the 1st of June to the 31st of August. Animals underwent an adaptation period of 15 days before starting the trial. Lactating buffaloes were maintained in open yards that allowed 15 m^2^/head and 80 cm manger. Animals were machine milked twice daily at 05.00 a.m. and 04.00 p.m. Thirty-two subjects at 63.00 ± 8.38 days in milk (DIM) were selected from a larger number of animals and randomly divided into two homogeneous groups, according to DIM, parity and milk yield. Dry Diet (DD) Group (n = 16; DIM = 66.81 ± 12.07; average milk yield of 13.45 ± 0.19 kg) received a diet characterized by dry forage and concentrate, whereas zero grazing (ZG) Group (n = 16; DIM = 59.19 ± 11.93; average milk yield of 13.43 ± 0.08 kg), fed a TMR on 30% green forage on the dry matter basis. *Sorghum bicolor* (drummondi variety) was cut twice daily at the boot stage and included in the diet, that was administered in the morning and in the afternoon. Similarly, also the diet of the DD Group was administered twice a day. Refusals were collected and weighed daily before the administration of the TMR. Furthermore, once a week a sample of single feedstuffs, refusals and TMR were collected and analyzed according to Association of Official Analytical Chemists (AOAC) in order to calculate dry matter intake of each group. Nutritional requirements were calculated according to previous studies^53^. Briefly, energy requirements, expressed as Milk Forage Units−MFU (1 MFU = 1700 kcal) were calculated according to the equations of INRA^54^(Eq. 1):

1 $$MFU_{req} = \, \left[ {\left( {1.4 \, + \, 0.006 \, \times \, kg \, \;live \, \;weight} \right) \, \times \, 1.1 \, + \, 0.44 \, \;MFU \, \times \, kg \, \;Energy\; \, Corrected\; \, Milk} \right]$$

Energy corrected milk (ECM = 740 kcal) was evaluated considering the formula from Campanile et al.^53^(Eq. 2):

2 $$ECM\; \, = \; \, milk \, \;yield \, * \, \left[ {\left\{ {fat \, \left( {g/kg} \right) \, {-} \, 40 \, + \, protein \, \left( {g/kg} \right){-} \, 31} \right\} \, * \, 0.01155} \right] \, + \, 1$$

Dry matter intake (DMI) was achieved by subtracting orts from the administered TMR before the subsequent administration, according to the following equation^53^(Eq. 3):

3 $$DMI \, = \, \left( {91 \, g \, * \, Metabolic \, \;Weight} \right) \, + \left( {0.27 \, kg \, * \, kg\; \, ECM} \right)$$

It is worth pointing out that the two diets had the same energy and protein content. The composition and the characteristics of the two diets are reported in Table 6.

**Table 6:** Total mixed ration (kg), chemical composition (% of dry matter, DM) and milk forage units (MFU) of the diets administered in buffaloes of Group zero grazing (ZG) and dry diet (DD).

Component	Feed (kg)	
	ZG	DD
*Sorghum bicolor*	26.0	–
Brewers grains	14.00	15.0

Component	Feed (kg)	
	ZG	DD
Maize silage	9.0	16.0
Hay	2.6	3.0
Corn flakes	2.5	2.2
Concentrate	2.1	2.0
Hydrogenated fat	0.15	0.25
Calcium carbonate	0.06	0.07
Sodium bicarbonate	0.04	0.06
Salt 1:3 (Ca/P)	0.03	0.02
**Chemical composition**		
DM (kg)	16.96	16.89
CP (%/DM)	14.57	14.50
EE (%/DM)	6.84	6.92
CF (%/DM)	18.57	18.64
NDF (%/DM)	35.72	36.06
ADF (%/DM)	20.56	22.86
Ashes (%/DM)	7.01	7.41
NSC (%/DM)	35.86	35.11
Starch (%/DM)	16.59	18.96
Calcium (%/DM)	0.85	0.83
Phospohorus (%/DM)	0.43	0.42
MFU (%/DM)	0.94	0.94

DM, dry matter; CP, crude protein; EE, ether extract; CF, crude fiber; NDF, neutral detergent fiber; ADF, acid detergent fiber; NSC, non-structural carbohydrates; MFU, milk forage units.

Finally, Body Condition Score (BCS) was assessed every 15 days by using the 1–9 scale, developed for beef cattle and modified for buffalo^55^.

### Milk and rumen fluid sampling

Throughout the experimental period (from the 1st of June to 31st of August), individual milk yield was recorded daily. Moreover, once a month, milk samples were individually collected during the morning milking routine to assess milk quality through infrared spectroscopy (Milkoscan, Ftplus 6000, Foss-Electric, Hillerød, Denmark) and somatic cell count (SCC, cells/mL) by Fossomatic FC (Foss Electric). 16 milk samples in each group ad for each sampling time were stored at − 80 °C and subsequently used to evaluate the metabolomic profile (see below). At the end of August (after 90 days from the start of the study), eight animals in each group were slaughtered and rumen content was individually sampled. The rumen fluid was stored at -80 °C until analyses.

### Metabolites extraction

Frozen milk samples were thawed at room temperature and vortexed for 30 s. Metabolites’ extraction was performed mixing 1 mL from each sample to 1 mL of methanol (Sigma-Aldrich, St. Louis, Missouri, USA) and vortexing for 30 s at room temperature. Mixture was stored at 4 °C for 10 min and then centrifuged at 12,000 rpm for 15 min at 4 °C. Supernatants were recovered and centrifuged again as previously described. The extracted supernatants were used to detect milk metabolites by LC–MS methodology. Samples of rumen fluid (15 mL) were centrifuged at 5000 rpm for 10 min. Then 1 mL of liquid was mixed with 1 mL of methanol and vortexed for 30 s at room temperature. After 10 min at − 20 °C, samples were centrifuged for 10 min at 15,000 rpm at 4 °C to allow the proteins precipitation. The supernatants were filtered (0.22 µm) and used for LC–MS analysis.

### LC–MS analysis

Detection of metabolites was done by using a high-performance liquid chromatograph (HPLC) 1260 Infinity Series (Agilent Technologies, Santa Clara, USA) equipped with a DAD (Diode Array Detector) system (Agilent Technologies, Santa Clara, USA) and coupled to a quadrupole-time of flight (Q-TOF) mass spectrometer (Agilent Technologies, Santa Clara, USA) with a Dual ESI source (Agilent Technologies, Santa Clara, USA). HPLC, UV and MS and parameters were calibrated by the Agilent Mass Hunter Data Acquisition Software, rev. B.05.01 (Agilent Technologies, Santa Clara, USA). Chromatographic separation was performed following the method reported by Comite et al.^56^ Among the detected molecules, only those with a mass error below 10 ppm and a sufficient score were reported (identification score > 80).

### Statistical analysis and compounds identification

Statistical analysis was carried out by using Mass Profiler Professional 13.1.1 software (Agilent Technologies, Santa Clara, CA, USA). Raw data were separated into two groups named DD Group and ZG Group forage based on the type of feed administered to the animals, choosing the ZG Group as a control group. Data were analyzed separately according to the three sampling times (June, July and August). Data were first subjected to principal components analysis (PCA) in order to highlight trends in the samples. Then, Student's t-test was performed to identify metabolites with significantly different levels (*p-*value < 0.05 and fold change > 2.0)^57,58^. Moreover, a longitudinal analysis of metabolic profiles was performed, separately for DD and ZG Group, choosing June sampling as control group. Multivariate and univariate analysis were carried out by using PCA and one-way ANOVA, screening metabolites by *p*-value < 0.05 and fold change > 2.0.

Compounds identification was carried out by comparison of spectrometric data using Milk Composition Database (MCDB), Bovine Metabolome Database (BMDB), Human Metabolome Database (HMDB), freely available databases and using authentic standards when available. Data on fat content, protein content, ECM, cheese yield, SCC and DMI were analyzed by analysis of variance (SPSS 26.0 for Windows 10, SPSS Inc., Chicago, IL), generalized linear mixed model, with diet (group) as the main factor and day of sampling as repeated measure. Milk yield was analyzed by analysis of variance, generalized linear mixed model, with diet (group) as the main factor and week as the repeated measure. To investigate the effect of DIM on milk yield and milk quality traits, two classes were created: 1 for buffaloes between 0 and 90 DIM, and 2 for buffaloes beyond 91 DIM. Contrasts were performed between and within classes, day of sampling was the repeated measure. Results are expressed as mean ± standard error (se). A statistically significant difference was accepted at *p* < 0.05.

### Ethics declarations

All procedures were approved by the Ethical Animal Care and Use Committee of the University of Naples Federico II, Italy (Protocol Number. 996072017). The study is reported in accordance with the ARRIVE guidelines (https://arriveguidelines.org).

## Supplementary Information

Supplementary Tables.

## Data Availability

The data that support the findings of this study are available from the corresponding author upon reasonable request.
