# Supplementary material for: Buffalo milk and rumen fluid metabolome are significantly affected by green feed
Source: Sci Rep. 2023 Jan 25;13:1381. doi: 10.1038/s41598-022-25491-w (PMC9877005; doi:10.1038/s41598-022-25491-w)
Supplement: Supplementary file 1 — Supplementary Tables. [file 41598_2022_25491_MOESM1_ESM.docx]

***Supplementary Information***

**Buffalo milk and rumen fluid metabolome are significantly affected by green feed**

**Neglia G^1^., Cotticelli A^1^., Vassetti A^2^., Matera R^1^., Staropoli A^2,3^., Vinale F^1,2*^., Salzano A^1^., Campanile G.^1^**

^1^Department of Veterinary Medicine and Animal Production, University of Naples “Federico II”, 80137 Naples, Italy; neglia@unina.it (G.N.); alessio.cotticelli@unina.it (A.C.); roberta.matera@unina.it (R.M.); angela.salzano@unina.it (A.S.); giucampa@unina.it (G.C.)

^2^Institute for Sustainable Plant Protection, National Research Council, 80055 Portici (NA), Italy; anastasia.vassetti@ipsp.cnr.it (A.V); alessia.staropoli@ipsp.cnr.it (A.S.)

^3^Department of Agricultural Sciences, University of Naples “Federico II”, 80055 Portici (NA), Italy;

*****Correspondence: frvinale@unina.it; Tel.: +39-0812539338

**TableS1.** Metabolites obtained from LC-MS data (positive mode), that are differentially accumulated in milk samples from buffaloes of DD Group (fed a total mixed ratio (TMR)) and ZG Group (fed TMR+30% of green forage) collected in June. Identifications were performed by comparing results with known compounds present in freely available electronic databases.

| Compound | Monoisotopic Mass (Da) | Retention Time (min) | p (Corr) | LnFC  DD vs. ZG |
| --- | --- | --- | --- | --- |
| C_7_H_13_NO_2_ | 143.0944 | 1.316333 | 0.002039 | -7.45708 |
| γ-Butyrobetaine | 145.1091 | 1.0739999 | 4.30E-18 | -18.1088 |
| C_6_H_2_NO_2_S_2_ | 183.9534 | 0.84623706 | 2.23E-05 | -11.437 |
| Acetylcarnitine | 203.1163 | 1.1329998 | 1.36E-24 | 21.28232 |
| Butyrylcarnitine | 231.1485 | 1.4879999 | 4.04E-25 | 22.13542 |
| 2-Methylbutyrylcarnitine | 245.1644 | 3.694 | 2.18E-23 | 19.65461 |
| Glycerophoshpocoline | 257.1036 | 1.2700003 | 2.18E-07 | -12.3487 |
| 2-Hexenoylcarnitine | 257.1664 | 3.9660013 | 1.48E-14 | 15.89597 |
| Hexanoylcarnitine | 259.1796 | 4.1210003 | 1.27E-12 | -15.6068 |
| C_19_H_21_NO_4_ | 327.1466 | 1.4220004 | 3.16E-10 | 14.15207 |
| C_22_H_43_NO | 337.3343 | 10.760997 | 3.02E-19 | 17.41308 |
| C_22_H_45_NO_2_ | 355.3486 | 10.757998 | 0.030449 | -3.53149 |
| C_17_H_19_N_3_O_2_S | 361.0937 | 1.0739998 | 2.00E-15 | 16.6681 |
| C_9_H_20_N_2_O_13_ | 364.0967 | 13.446006 | 5.86E-24 | 20.47424 |
| C_9_H_20_N_2_O_13_ | 364.0973 | 0.9689999 | 1.83E-24 | 21.43499 |
| C_9_H_20_N_2_O_13_ | 364.0979 | 1.0779996 | 7.20E-25 | 21.88393 |
| C_14_H_20_O_11_ | 364.1003 | 4.218001 | 0.003133 | 7.484329 |
| C_16_H_19_N_3_O_5_S | 365.1014 | 1.107 | 5.68E-05 | -10.4531 |
| C_12_H_16_N_2_O_12_ | 380.0714 | 0.91600025 | 1.36E-24 | 22.40428 |
| C_17_H_16_O_10_ | 380.0742 | 4.0120006 | 1.07E-17 | 18.12295 |
| C_11_H_16_N_3_O_12_ | 382.0719 | 0.9139997 | 5.81E-23 | 19.66598 |
| C_26_H_37_Cl | 384.2586 | 3.945 | 0.010435 | 5.238881 |
| N.a. | 425.8936 | 0.84730744 | 2.79E-05 | -9.09479 |
| C_22_H_46_NO_7_P | 467.3047 | 6.2359977 | 3.49E-14 | 15.5879 |
| **Continued** |  |  |  |  |
| **Compound** | **Monoisotopic Mass (Da)** | **Retention Time (min)** | **p (Corr)** | **LnFC**  **DD vs. ZG** |
| C_16_H_31_N_17_O | 477.2885 | 6.6979985 | 1.44E-22 | -18.9163 |
| C_23_H_44_NO_7_P | 477.2891 | 6.697002 | 1.68E-22 | -18.8592 |
| C_23_H_46_NO_7_P | 479.3048 | 7.3269453 | 3.50E-11 | 13.86927 |
| N.a. | 481.3991 | 10.471 | 1.31E-21 | 18.20975 |
| C_17_H_37_N_17_O | 495.3361 | 6.977998 | 5.15E-18 | 16.36692 |
| C_24_H_50_NO_7_P | 495.3377 | 7.171002 | 1.36E-24 | 21.21314 |
| C_26_H_52_NO_7_P | 521.3522 | 7.4989967 | 2.87E-23 | 19.58995 |
| C_17_H_37_N_20_ | 521.3528 | 7.309692 | 2.31E-04 | 8.051291 |
| C_26_H_54_NO_7_P | 523.3668 | 8.714001 | 5.12E-12 | 14.75573 |
| N.a. | 525.4268 | 10.391999 | 2.22E-21 | 18.03185 |
| N.a. | 555.8524 | 0.8532174 | 7.03E-05 | -8.94925 |
| C_22_H_24_N_21_ | 582.2515 | 5.6330004 | 3.59E-16 | 15.53616 |
| C_22_H_31_N_12_O_15_ | 703.2044 | 1.0886275 | 3.55E-14 | 15.56817 |
| C_21_H_42_N_2_O_24_ | 706.2132 | 0.97500026 | 3.78E-24 | 21.21644 |
| C_21_H_42_N_2_O_24_ | 706.2145 | 1.0989995 | 3.96E-14 | 17.86918 |
| C_21_H_42_N_2_O_24_ | 706.215 | 1.0989995 | 3.96E-14 | 17.86912 |
| C_29_H_32_N_5_O_17_ | 722.1819 | 0.9099999 | 4.30E-18 | 15.37066 |
| C_26_H_32_N_8_O_16_S | 744.1664 | 0.9169999 | 9.87E-19 | -15.9103 |
| N.a. | 768.5166 | 3.9960003 | 6.63E-05 | -7.58207 |
| N.a. | 805.4072 | 4.4859996 | 6.92E-04 | -5.98812 |
| N.a. | 807.5377 | 5.1029987 | 0.046608 | -4.15043 |
| N.a. | 914.2022 | 1.0482858 | 0.003815 | 7.672951 |
| N.a. | 961.67 | 4.128999 | 2.64E-21 | 17.44395 |
| N.a. | 1014.5603 | 3.990999 | 5.18E-04 | 8.430172 |
| N.a. | 1064.294 | 1.0620002 | 3.96E-14 | 16.88831 |
| N.a. | 1226.7131 | 4.1559987 | 9.32E-10 | 12.84251 |
| N.a. | 1227.8126 | 4.1429996 | 9.56E-09 | -11.5006 |
| N.a. | 1243.8081 | 4.166 | 3.66E-17 | -16.3656 |
| N.a. | 1259.8003 | 4.173998 | 2.67E-07 | -11.2193 |
| N.a. | 1275.8007 | 3.727999 | 7.54E-10 | 11.91177 |
| N.a. | 1289.0618 | 4.9899983 | 0.038591 | -4.10426 |
| N.a. | 1406.408 | 1.0580001 | 1.20E-19 | 18.00962 |
| N.a. | 1406.4084 | 1.0580001 | 1.20E-19 | 18.00962 |
| N.a. | 1406.4094 | 1.0580001 | 1.20E-19 | 18.00962 |
| N.a. | 1649.7491 | 4.375 | 0.039255 | -3.50774 |
| N.a. | 1732.3184 | 4.1269994 | 3.70E-04 | -6.50509 |
| N.a. | 1748.6978 | 4.0889993 | 0.020793 | -4.15099 |
| N.a. | 2090.8213 | 3.9160001 | 0.00564 | -4.38107 |
| N.a. | 2123.1316 | 4.087999 | 3.05E-05 | 7.899328 |
| N.a. | 2123.1833 | 4.278 | 5.24E-15 | 15.06918 |
| N.a. | 2123.6826 | 4.278 | 1.08E-16 | 15.74817 |
| N.a. | 2148.3486 | 5 | 0.003582 | -6.78119 |
| N.a. | 2210.2698 | 3.7200007 | 4.91E-09 | 11.57197 |
| N.a. | 2236.2153 | 4.136001 | 4.61E-20 | 18.13292 |
| **Continued** |  |  |  |  |
| **Compound** | **Monoisotopic Mass (Da)** | **Retention Time (min)** | **p (Corr)** | **LnFC**  **DD vs. ZG** |
| N.a. | 2363.611 | 4.11 | 4.14E-07 | -10.0643 |
| N.a. | 2363.6943 | 4.108001 | 4.12E-07 | -10.1397 |
| N.a. | 2364.6978 | 4.0869985 | 7.70E-04 | 6.891056 |
| N.a. | 2378.3684 | 3.837999 | 1.27E-08 | 11.84865 |
| N.a. | 2540.4353 | 3.6520002 | 1.47E-22 | 18.97393 |
| N.a. | 2541.436 | 3.653 | 5.15E-20 | 16.6996 |
| N.a. | 2554.4502 | 3.7559986 | 0.008437 | 6.099718 |
| N.a. | 2589.1436 | 3.7509997 | 5.05E-07 | 10.07036 |
| N.a. | 2864.5415 | 3.7089999 | 3.49E-14 | 14.47743 |
| N.a. | 2887.8335 | 4.071999 | 0.009411 | -4.88555 |
| N.a. | 2914.4922 | 4.0950007 | 0.005675 | -4.41916 |
| N.a. | 2914.4941 | 4.11 | 0.005419 | -4.51371 |
| N.a. | 3125.684 | 4.492998 | 0.008826 | 4.879009 |
| N.a. | 3229.596 | 5.1 | 0.020793 | -5.72142 |
| N.a. | 3352.8462 | 4.395998 | 2.79E-08 | 11.04398 |
| N.a. | 3598.6377 | 3.9990003 | 0.021072 | -4.13911 |
| N.a. | 3927.3672 | 4.57 | 0.043688 | -4.71822 |
| N.a. | 3968.9417 | 3.8509996 | 0.008362 | -4.25051 |
| N.a. | 3980.1602 | 4.378999 | 1.64E-13 | 17.6712 |
| N.a. | 3981.1765 | 4.3779993 | 7.78E-24 | 19.81799 |
| N.a. | 4003.1792 | 4.3926196 | 6.79E-10 | -13.236 |
| N.a. | 4019.1057 | 4.394462 | 6.47E-04 | -7.46112 |
| N.a. | 4049.91 | 3.8799994 | 0.002341 | -5.09068 |
| N.a. | 4117.2495 | 4.323999 | 6.81E-16 | 16.18638 |
| N.a. | 4118.255 | 4.3229985 | 2.85E-15 | 15.31988 |
| N.a. | 4124.3555 | 4.3489995 | 3.17E-05 | 9.424458 |
| N.a. | 4124.8613 | 4.346 | 5.52E-06 | 10.16233 |
| N.a. | 4139.2334 | 4.347 | 2.02E-05 | -9.11418 |
| N.a. | 4187.279 | 4.6494083 | 5.68E-05 | -7.88961 |
| N.a. | 4187.3457 | 4.64763 | 5.61E-05 | -8.01942 |
| N.a. | 4187.3896 | 4.6790004 | 1.22E-04 | -7.54793 |
| N.a. | 4214.192 | 4.5159993 | 0.002375 | -6.08964 |
| N.a. | 4229.055 | 4.181 | 0.00119 | 6.795452 |
| N.a. | 4245.353 | 4.278 | 8.60E-20 | 17.59212 |
| N.a. | 4247.3545 | 4.2790003 | 3.45E-21 | 17.16512 |
| N.a. | 4268.341 | 4.275001 | 5.66E-05 | 7.737533 |
| N.a. | 4269.702 | 4.5920014 | 5.25E-09 | -13.1291 |
| N.a. | 4284.3125 | 4.298998 | 0.007132 | -5.16827 |
| N.a. | 4309.02 | 4.238999 | 1.00E-21 | 17.64671 |
| N.a. | 4442.371 | 4.4290004 | 0.003441 | -5.66633 |
| N.a. | 4679.248 | 4.185999 | 1.12E-12 | 14.09685 |
| N.a. | 4679.4775 | 4.191001 | 1.27E-14 | 14.43852 |
| N.a. | 4727.368 | 4.126 | 6.77E-06 | -8.92564 |
| N.a. | 4744.5957 | 4.582201 | 0.001901 | -8.60272 |
| **Continued** |  |  |  |  |
| **Compound** | **Monoisotopic Mass (Da)** | **Retention Time (min)** | **p (Corr)** | **LnFC**  **DD vs. ZG** |
| N.a. | 4807.3345 | 4.128999 | 6.87E-23 | 18.85234 |
| N.a. | 4830.3213 | 4.143134 | 0.015331 | -5.83205 |
| N.a. | 5131.4434 | 4.2020016 | 7.28E-07 | -10.6473 |
| N.a. | 5132.444 | 4.171999 | 1.72E-06 | 9.338116 |
| N.a. | 6139.0557 | 4.1439996 | 0.006407 | -5.02338 |
| N.a. | 6219.0254 | 4.166 | 9.13E-10 | -12.8649 |

Milk Composition Database (MCDB) and Bovine Metabolome Database (BMDB) were used for the identification. n.a. = compounds for which it was not possible to assign a name and/or a molecular formula. p(Corr) = corrected p-value obtained after application of Benjamini-Hochberg procedure. LnFC = Natural logarithm of Fold Change.

**TableS2** Metabolites obtained from LC-MS data (positive mode), that are differentially accumulated in milk samples from buffaloes of DD Group (fed a total mixed ratio (TMR)) and ZG Group (fed TMR+30% of green forage) collected in July. Identifications were performed by comparing results with known compounds present in freely available electronic databases.

| Compound | Monoisotopic Mass (Da) | Retention Time (min) | p (Corr) | LnFC  DD vs. ZG | |  |
| --- | --- | --- | --- | --- | --- | --- |
| γ-Butyrobetaine | 145.1099 | 1.0947931 | 0.002618 | -7.8222 | |  |
| δ-Valerobetaine | 159.1254 | 1.0077583 | 0.042389 | -2.98936 | |  |
| L-Carnitine | 161.1045 | 1.0410691 | 0.036103 | -3.20882 | |  |
| C_6_NO_3_S_2_ | 197.9328 | 0.84175915 | 0.04049 | -3.07697 | |  |
| Acetylcarnitine | 203.1158 | 1.0362759 | 0.042276 | -2.96594 | |  |
| N.a. | 203.1164 | 1.3903451 | 0.04049 | -3.04639 | |  |
| Propionylcarnitine | 217.1318 | 1.1379608 | 1.02E-08 | -14.4192 | |  |
| Butyrylcarnitine | 231.1482 | 1.5236902 | 0.045992 | -2.93003 | |  |
| 2-Methylbutyrylcarnitine | 245.1642 | 3.7455182 | 0.04049 | -3.0367 | |  |
| C_13_H_19_N_5_ | 245.1646 | 3.74 | 1.46E-18 | 18.08596 | |  |
| C12 H17 O6 | 257.1023 | 0.89100015 | 1.08E-16 | 16.18497 | |  |
| Glycerophoshpocoline | 257.1031 | 1.2308275 | 0.04049 | -3.10791 | |  |
| C_8_H_14_N_3_O_7_P | 295.0575 | 0.88944805 | 0.036103 | -3.25272 | |  |
| C_17_H_34_O_4_ | 302.2456 | 9.335003 | 5.66E-04 | -6.56747 | |  |
| C_9_H_20_N_2_O_13_ | 364.0968 | 13.476144 | 0.021755 | -3.56129 | |  |
| C_10_H_16_N_6_O_9_ | 364.0991 | 0.9559656 | 0.036103 | -3.25123 | |  |
| C_14_H_20_O_11_ | 364.1007 | 3.7872593 | 1.63E-17 | 17.31521 | |  |
| C_20_H_11_N_7_O | 365.1023 | 0.97100025 | 1.25E-23 | -23.9815 | |  |
| N.a. | 365.1038 | 1.2571727 | 0.04049 | -3.04639 | |  |
| N.a. | 365.1038 | 1.2840003 | 0.04049 | -3.04639 | |  |
| C_13_H_12_N_6_O_8_ | 380.072 | 0.90534484 | 0.036103 | -3.25176 | |  |
| C_9_H_20_N_2_O_12_S | 380.0737 | 4.2340007 | 0.036103 | -3.61962 | |  |
| C_17_H_16_O_10_ | 380.0742 | 3.9940014 | 6.06E-19 | -22.778 | |  |
| C_17_H_16_O^10^ | 380.0743 | 3.9642456 | 0.001119 | -9.09615 | |  |
| C_11_H_16_N_3_O_12_ | 382.073 | 0.9069997 | 5.24E-18 | 17.50587 | |  |
| N.a. | 382.0746 | 1.227 | 0.04049 | -3.04639 | |  |
| C_26_H_37_Cl | 384.2581 | 3.947539 | 1.14E-06 | -10.6669 | |  |
| N.a. | 412.7275 | 3.851999 | 0.017731 | -4.05824 | |  |
| N.a. | 425.8939 | 0.8339998 | 0.031604 | -4.9977 | |  |
| N.a. | 441.871 | 0.84271437 | 0.019567 | -4.34597 | |  |
| C_22_H_46_NO_7_P | 467.3041 | 6.2585864 | 0.036103 | -3.22999 | |  |
| C_14_H_29_N_20_ | 477.2884 | 6.701846 | 1.51E-11 | -17.2092 | |  |
| C_23_H_46_NO_7_P | 479.3038 | 7.233001 | 0.007036 | -5.18778 | |  |
| C_27_H_43_O_7_ | 479.3042 | 7.4339256 | 0.040343 | -3.13281 | |  |
| C_23_H_45_C_l2_N_6_S | 507.2804 | 3.9830012 | 1.81E-06 | -12.0781 | |  |
| C_15_H_25_N_11_O_9_S | 535.1562 | 13.592998 | 0.008189 | -5.28632 | |  |
| N.a. | 571.8296 | 0.84373975 | 0.04049 | -4.55282 | |  |
| C_17_H_36_N_18_O_5_ | 572.3112 | 3.710999 | 0.017731 | -4.23745 | |  |
| N.a. | 587.8071 | 0.8473845 | 0.040343 | -6.16704 | |  |
| C_23_H_19_N_19_OS | 609.1764 | 13.708005 | 3.24E-05 | -12.6255 | |  |
| C_25_H_50_N_6_O_14_ | 658.3395 | 4.1480007 | 0.017731 | -4.09798 | |  |
| C_24_H_40_N_3_O_21_ | 706.2159 | 0.97100013 | 2.67E-20 | 19.95616 | |  |
| C_22_H_38_N_6_O_20_ | 706.2171 | 0.98168993 | 0.003603 | -6.11263 | |  |
| C_21_H_43_N_2_O_24_ | 707.221 | 0.9757236 | 0.034941 | -3.29503 | |  |
| C_25_H_57_N_17_O_3_S_2_ | 707.4269 | 3.8990006 | 0.005541 | -6.03394 | |  |
| C_22_H_36_N_5_O_22_ | 722.178 | 1.2210003 | 1.02E-08 | -17.0873 | |  |
|  |  |  |  |  | |  |
| **Continued** |  |  |  |  | |  |
| **Compound** | **Monoisotopic Mass (Da)** | **Retention Time (min)** | **p (Corr)** | **LnFC**  **DD vs. ZG** | |  |
|  |  |  |  |  | |  |
| N.a. | 722.1836 | 1.6059994 | 0.04049 | -3.04639 | |  |
| C_29_H_22_N_16_O_8_ | 722.1854 | 0.90399975 | 2.92E-17 | -18.5815 | |  |
| N.a. | 768.5167 | 3.950999 | 0.003724 | 6.633111 | |  |
| N.a. | 823.8778 | 4.3229985 | 0.04049 | -3.04639 | |  |
| N.a. | 823.932 | 4.319 | 0.04049 | -3.04639 | |  |
| N.a. | 849.3911 | 4.2620006 | 0.04049 | -3.04639 | |  |
| N.a. | 849.4084 | 4.272998 | 0.04049 | -3.04639 | |  |
| N.a. | 849.4243 | 4.2720013 | 0.04049 | -3.04639 | |  |
| N.a. | 897.543 | 4.233001 | 0.017731 | -4.1823 | |  |
| N.a. | 920.731 | 4.9376955 | 0.04049 | -3.04639 | |  |
| N.a. | 936.5344 | 3.8990006 | 0.017731 | -3.98836 | |  |
| N.a. | 961.6714 | 4.127135 | 0.031098 | -2.98384 | |  |
| N.a. | 961.9637 | 4.135105 | 0.04049 | -3.04639 | |  |
| N.a. | 962.0089 | 4.1470013 | 0.04049 | -3.04639 | |  |
| N.a. | 1014.5612 | 3.986 | 0.014433 | 6.070646 | |  |
| N.a. | 1026.4912 | 4.1699986 | 1.74E-04 | -6.9372 | |  |
| N.a. | 1064.2944 | 1.087 | 7.81E-06 | -11.8115 | |  |
| N.a. | 1064.2954 | 1.2320184 | 0.044183 | -5.94249 | |  |
| N.a. | 1064.2961 | 1.1559998 | 4.00E-22 | -21.5205 | |  |
| N.a. | 1064.2961 | 1.2459998 | 3.67E-05 | -11.7706 | |  |
| N.a. | 1065.2972 | 1.0934683 | 3.21E-05 | -10.6477 | |  |
| N.a. | 1162.6166 | 3.5619984 | 0.017731 | -5.04085 | |  |
| N.a. | 1236.672 | 4.2819996 | 1.45E-08 | -9.91836 | |  |
| N.a. | 1259.8009 | 4.128999 | 1.74E-04 | -10.0544 | |  |
| N.a. | 1289.2028 | 4.9343624 | 0.04049 | -3.04639 | |  |
| N.a. | 1406.4125 | 1.1059613 | 0.017731 | 6.302727 | |  |
| N.a. | 1743.0537 | 3.7040007 | 0.018048 | -4.10697 | |  |
| N.a. | 1848.0262 | 3.6559987 | 0.017731 | -4.01248 | |  |
| N.a. | 1950.5916 | 4.3185525 | 0.031604 | -4.59129 | |  |
| N.a. | 1951.0896 | 4.3130016 | 6.15E-13 | -12.6455 | |  |
| N.a. | 1990.5942 | 4.3739996 | 0.005936 | -4.85653 | |  |
| N.a. | 1991.0934 | 4.3769994 | 0.017731 | -4.01365 | |  |
| N.a. | 2047.1633 | 3.8300014 | 0.017731 | -4.08409 | |  |
| N.a. | 2059.645 | 4.323999 | 0.002792 | -5.25296 | |  |
| N.a. | 2100.0037 | 4.134001 | 1.53E-04 | -10.0989 | |  |
| N.a. | 2123.132 | 4.085142 | 0.02614 | -5.34213 | |  |
| N.a. | 2123.6833 | 4.2699986 | 2.61E-14 | 14.0728 | |  |
| N.a. | 2148.3406 | 4.942001 | 0.04049 | -3.04639 | |  |
| N.a. | 2149.0308 | 4.945 | 0.04049 | -3.04639 | |  |
| N.a. | 2153.315 | 5.0120006 | 0.04049 | -3.04639 | |  |
| N.a. | 2224.2852 | 3.7990017 | 0.017731 | -4.04607 | |  |
| N.a. | 2236.216 | 4.132484 | 0.023102 | -2.87762 | |  |
| N.a. | 2363.693 | 4.0899987 | 0.047721 | 4.24225 | |  |
| N.a. | 2404.1787 | 4.1304774 | 0.031098 | -4.64144 | |  |
| N.a. | 2579.3584 | 4.0861936 | 0.040657 | -4.23225 | |  |
| N.a. | 2826.5537 | 4.240999 | 6.90E-05 | -7.13775 | |  |
| N.a. | 2878.5542 | 3.805 | 0.017731 | -4.1398 | |  |
| N.a. | 2914.6304 | 4.055 | 0.04049 | -3.04639 | |  |
| N.a. | 3125.7043 | 4.4929986 | 0.001165 | -6.25703 | |  |
| N.a. | 3222.5823 | 4.934 | 2.53E-05 | -10.6871 | |  |
|  |  |  |  |  | |  |
| **Continued** |  |  |  |  | |  |
| **Compound** | **Monoisotopic Mass (Da)** | **Retention Time (min)** | **p (Corr)** | | **LnFC**  **DD vs. ZG** | |
|  |  |  |  |  | |  |
|  |  |  |  |  | |  |
| N.a. | 3297.5186 | 4.184999 | 0.031098 | -3.5534 | |  |
| N.a. | 3599.651 | 3.9339986 | 0.012707 | -4.54048 | |  |
| N.a. | 3838.4536 | 4.612 | 0.04049 | -3.04639 | |  |
| N.a. | 3901.1638 | 4.3209996 | 1.12E-06 | -9.46013 | |  |
| N.a. | 3924.4568 | 4.3959985 | 0.04049 | -3.04639 | |  |
| N.a. | 3969.952 | 3.796999 | 0.017731 | -4.08648 | |  |
| N.a. | 3980.17 | 4.3717904 | 0.02653 | -3.07777 | |  |
| N.a. | 3997.1826 | 4.305 | 0.001171 | -6.10785 | |  |
| N.a. | 4012.1992 | 4.2440014 | 0.002721 | -5.67683 | |  |
| N.a. | 4044.3955 | 4.314001 | 0.04049 | -3.04639 | |  |
| N.a. | 4044.9004 | 4.317001 | 0.017731 | -4.04083 | |  |
| N.a. | 4049.9165 | 3.819 | 0.017731 | -4.06419 | |  |
| N.a. | 4178.0127 | 3.7440012 | 0.017731 | -4.07944 | |  |
| N.a. | 4270.091 | 4.57 | 0.017731 | -4.04233 | |  |
| N.a. | 4309.022 | 4.2357926 | 0.017731 | -3.27604 | |  |
| N.a. | 4482.466 | 4.491754 | 0.034377 | -4.27204 | |  |
| N.a. | 4570.4487 | 4.315605 | 0.047048 | -4.34322 | |  |
| N.a. | 4679.245 | 4.1869984 | 1.25E-23 | -16.8415 | |  |
| N.a. | 4680.2617 | 4.1910014 | 7.78E-08 | 11.49812 | |  |
| N.a. | 4807.3413 | 4.1261387 | 0.01061 | -3.55583 | |  |
| N.a. | 4808.3423 | 4.1267834 | 0.001748 | -6.3301 | |  |
| N.a. | 4830.323 | 4.122001 | 1.68E-06 | -8.72508 | |  |
| N.a. | 4844.102 | 4.5510006 | 0.017731 | -4.12314 | |  |
| N.a. | 4844.4097 | 4.558999 | 0.031098 | -3.56942 | |  |
| N.a. | 4844.683 | 4.5540004 | 0.017731 | -4.11452 | |  |
| N.a. | 5131.443 | 4.1699986 | 4.20E-17 | -14.2515 | |  |
| N.a. | 5132.4536 | 4.176 | 0.04049 | 4.622545 | |  |
| N.a. | 5248.1055 | 4.5429997 | 0.04049 | -3.04639 | |  |
| N.a. | 5248.4346 | 4.5540004 | 0.017731 | -4.08059 | |  |
| N.a. | 5890.59 | 4.5429997 | 0.017731 | -4.02824 | |  |
| N.a. | 6579.4346 | 4.613999 | 0.017731 | -4.15846 | |  |

Milk Composition Database (MCDB) and Bovine Metabolome Database (BMDB) were used for the identification. n.a. = compounds for which it was not possible to assign a name and/or a molecular formula. p(Corr) = corrected p-value obtained after application of Benjamini-Hochberg procedure. LnFC = Natural logarithm of Fold Change.

**TableS3** Metabolites obtained from LC-MS data (positive mode), that are differentially accumulated in milk samples from buffaloes of DD Group (fed a total mixed ratio (TMR)) and ZG Group (fed TMR+30% of green forage) collected in August. Identifications were performed by comparing results with known compounds present in freely available electronic databases.

| Compound | Monoisotopic Mass (Da) | Retention Time (min) | p (Corr) | LnFC  DD vs. ZG |
| --- | --- | --- | --- | --- |
| C_15_H_13_NO | 103.0991 | 0.9913722 | 4.39E-10 | -19.0715 |
| C5 H13 N O | 103.0994 | 0.99799985 | 8.70E-26 | -31.4894 |
| N.a. | 103.0998 | 1.546 | 7.50E-20 | -14.6672 |
| C_7_H_13_NO_2_ | 143.0949 | 1.1044 | 4.03E-06 | -9.55257 |
| C_7_H_13_NO_2_ | 143.0956 | 1.2820344 | 1.03E-04 | -9.49925 |
| γ-Butyrobetaine | 145.1098 | 1.0924994 | 5.05E-15 | -16.2603 |
| C_5_H_13_N_4_O | 145.1098 | 1.09 | 0.019047 | 3.388227 |
| C_5_H_13_N_4_O | 145.1098 | 1.09 | 0.019047 | 3.388227 |
| C_5_H_13_N_4_O | 145.1099 | 1.09 | 0.019047 | 3.388227 |
| C_5_H_13_N_4_O | 145.11 | 1.09 | 0.019047 | 3.388227 |
| δ-Valerobetaine | 159.1252 | 0.9993226 | 2.84E-19 | -14.3323 |
| L-Carnitine | 161.1047 | 1.0292253 | 2.35E-19 | -14.6969 |
| C_6_NO_3_S_2_ | 197.9326 | 0.83583874 | 7.50E-20 | -14.7636 |
| Acetylcarnitine | 203.1157 | 1.0180323 | 9.22E-20 | -14.6381 |
| N.a. | 203.1165 | 1.6146128 | 7.50E-20 | -14.6672 |
| Propionylcarnitine | 217.1302 | 1.1779996 | 5.96E-08 | -10.7945 |
| C_8_H_17_N_4_O_3_ | 217.1306 | 1.192 | 4.11E-05 | 4.767893 |
| C_11_H_15_N_5_ | 217.1325 | 1.3061613 | 1.23E-20 | -15.5337 |
| Butyrylcarnitine | 231.148 | 1.4980001 | 2.98E-09 | 7.460587 |
| N.a. | 231.1484 | 1.8979995 | 7.50E-20 | -14.6672 |
| 2-Methylbutyrylcarnitine | 245.1644 | 3.662001 | 1.31E-05 | 5.092634 |
| C_16_H_17_N_5_ | 279.1486 | 3.945528 | 2.14E-07 | -10.218 |
| C_18_H_35_NO | 281.2704 | 11.109453 | 6.06E-11 | -18.035 |
| C_8_H_14_N_3_O_7_P | 295.0573 | 0.8823546 | 2.13E-19 | -14.7528 |
| C_17_H_34_O_4_ | 302.2464 | 9.403965 | 2.05E-06 | -13.0701 |
| N.a. | 327.1467 | 2.9089994 | 7.50E-20 | -14.6672 |
| C_9_N_2_O_6_S_3_ | 327.8915 | 0.8388538 | 7.17E-13 | -19.2789 |
| N.a. | 337.3333 | 11.302001 | 7.50E-20 | -14.6672 |
| C_20_H_41_N_4_ | 337.3334 | 10.832 | 4.26E-05 | 4.743446 |
| C_20_H_41_N_4_ | 337.3335 | 10.832 | 4.26E-05 | 4.743446 |
| C_20_H_41_N_4_ | 337.3335 | 10.832 | 4.26E-05 | 4.743446 |
| C_20_H_41_N_4_ | 337.3336 | 10.839997 | 0.012498 | 3.649581 |
| N.a. | 337.3337 | 10.609997 | 7.50E-20 | -14.6672 |
| C_20_H_41_N_4_ | 337.3338 | 12.135001 | 1.65E-19 | -18.8813 |
| C_20_H_41_N_4_ | 337.334 | 10.832 | 4.69E-05 | 4.716665 |
| C_20_H_41_N_4_ | 337.3341 | 10.832 | 4.26E-05 | 4.743446 |
| N.a. | 364.0969 | 12.778005 | 7.50E-20 | -14.6672 |
| C_9_H_20_N_2_O_13_ | 364.0971 | 13.520068 | 2.16E-16 | -24.2382 |
|  |  |  |  |  |
| **Continued** |  |  |  |  |
| **Compound** | **Monoisotopic Mass (Da)** | **Retention Time (min)** | **p (Corr)** | **LnFC**  **DD vs. ZG** |
|  |  |  |  |  |
| C_10_H_16_N_6_O_9_ | 364.0994 | 0.9355155 | 1.60E-19 | -14.3775 |
| C_9_H_20_N_2_O_13_ | 364.101 | 1.3028712 | 6.20E-18 | -16.4479 |
| N.a. | 364.1014 | 1.4799997 | 7.50E-20 | -14.6672 |
| C_18_H_9_N_10_ | 365.1026 | 0.95999956 | 2.50E-07 | 6.22836 |
| C_18_H_9_N_10_ | 365.1028 | 1.1089998 | 1.35E-16 | -14.0846 |
| N.a. | 365.1038 | 1.2757093 | 7.50E-20 | -14.6672 |
| N.a. | 365.104 | 1.3605413 | 7.50E-20 | -14.6672 |
| N.a. | 367.4231 | 8.912999 | 7.50E-20 | -14.6672 |
| C_13_H_12_N_6_O_8_ | 380.0714 | 0.8988386 | 3.45E-21 | -16.6328 |
| C_17_H_16_O_10_ | 380.0743 | 3.9932652 | 1.96E-11 | -23.7065 |
| N.a. | 380.0749 | 1.472 | 7.50E-20 | -14.6672 |
| N.a. | 380.075 | 1.3670002 | 7.50E-20 | -14.6672 |
| C_11_H_16_N_3_O_12_ | 382.0713 | 0.97100025 | 1.65E-10 | -12.2749 |
| C_11_H_16_N_3_O_12_ | 382.0725 | 0.90290314 | 7.50E-20 | -15.1303 |
| N.a. | 382.0746 | 1.5289998 | 7.50E-20 | -14.6672 |
| N.a. | 382.0751 | 1.2482252 | 7.50E-20 | -14.6672 |
| C_15_H_36_N_4_O_7_ | 384.2589 | 3.9342725 | 8.83E-09 | -13.3249 |
| C_22_H_37_N_3_OS | 391.2666 | 4.0729985 | 2.54E-15 | -17.1253 |
| C_22_H_49_N_6_O | 413.3969 | 10.743 | 6.02E-18 | -15.1988 |
| C_25_H_54_N_5_ | 424.4383 | 8.222996 | 3.26E-17 | -15.3171 |
| N.a. | 425.8929 | 0.83410543 | 1.74E-08 | -13.4476 |
| C_13_H_32_N_7_O_9_ | 430.2249 | 3.9380014 | 9.96E-15 | -13.5614 |
| C_25_H_54_N_5_O | 440.4331 | 8.334 | 3.26E-17 | -15.3168 |
| N.a. | 441.8706 | 0.8389181 | 1.16E-18 | -15.3214 |
| N.a. | 457.8484 | 0.8401701 | 1.30E-13 | -17.2783 |
| C_26_H_43_O_7_ | 467.3011 | 6.2700005 | 0.0012 | 3.673312 |
| C_26_H_43_O_7_ | 467.3013 | 6.2675805 | 7.50E-20 | -14.7911 |
| C_27_H_41_O_7_ | 477.2848 | 6.7319984 | 7.83E-04 | 3.833391 |
| C_27_H_41_O_7_ | 477.2854 | 6.728612 | 1.39E-19 | -15.1354 |
| C_27_H_43_O_7_ | 479.3007 | 7.4394827 | 1.17E-16 | -16.043 |
| N.a. | 495.332 | 7.472 | 7.50E-20 | -14.6672 |
| N.a. | 495.3321 | 7.4190025 | 7.50E-20 | -14.6672 |
| C_28_H_47_O_7_ | 495.3326 | 7.214162 | 1.03E-19 | -14.8921 |
| C_14_H_39_N_16_O_4_ | 495.3345 | 7.0299993 | 1.96E-07 | -10.2957 |
| C_15_H_42_N_15_O_2_S | 496.3353 | 7.2160025 | 0.014733 | 3.548831 |
| C_18_H_37_NO_13_S | 507.1981 | 2.2799995 | 4.46E-22 | -15.2917 |
| C_24_H_49_N_3_S_4_ | 507.2806 | 3.9725924 | 2.39E-11 | -16.1723 |
| C_15_H_43_N_12_O_8_ | 519.3321 | 6.800998 | 0.003051 | 3.353293 |
| C_26_H_50_NO_7_P | 519.3323 | 6.800031 | 9.41E-20 | -14.7765 |
| C_16_H_41_N_16_O_4_ | 521.3469 | 7.4379964 | 0.006679 | -5.66217 |
| N.a. | 521.3476 | 7.7940025 | 7.50E-20 | -14.6672 |
|  |  |  |  |  |
|  |  |  |  |  |
| **Continued** |  |  |  |  |
| **Compound** | **Monoisotopic Mass (Da)** | **Retention Time (min)** | **p (Corr)** | **LnFC**  **DD vs. ZG** |
| N.a. | 521.3478 | 7.7590017 | 7.50E-20 | -14.6672 |
| C_28_H_47_N_3_O_6_ | 521.3479 | 7.3670006 | 1.92E-07 | -9.90264 |
| C_19_H_39_N_17_O | 521.348 | 7.554258 | 7.50E-20 | -14.981 |
| C_28_H_47_N_3_O_6_ | 521.348 | 7.3670006 | 1.92E-07 | -9.9033 |
| C_22_H_15_N_16_O_2_ | 535.1564 | 13.601004 | 1.10E-06 | -10.0261 |
| C_15_H_4_Cl_3_N_2_O_9_S_3_ | 556.8161 | 4.036001 | 3.11E-11 | -11.9684 |
| N.a. | 571.8298 | 0.8414879 | 6.84E-13 | -19.4961 |
| C_23_H_30_N_14_O_5_ | 582.2497 | 5.64649 | 2.28E-12 | -18.9294 |
| N.a. | 609.1774 | 10.839997 | 7.50E-20 | -14.6672 |
| N.a. | 609.1818 | 10.519001 | 7.50E-20 | -14.6672 |
| C_28_H_39_N_17_ | 613.3583 | 4.1370006 | 0.008388 | -4.99872 |
| C_24_H_54_N_2_O_18_ | 658.3382 | 4.132002 | 2.48E-15 | -13.655 |
| N.a. | 666.2195 | 1.2690006 | 7.50E-20 | -14.6672 |
| C_21_H_42_N_2_O_24_ | 706.215 | 0.97538716 | 2.45E-20 | -15.1849 |
| C_29_H_40_NO_19_ | 706.2199 | 3.928001 | 4.66E-06 | 5.493465 |
| C_29_H_40_NO_19_ | 706.2199 | 3.9533231 | 7.50E-20 | -15.8228 |
| C_29_H_40_NO_19_ | 706.2202 | 3.928001 | 3.34E-06 | 5.561882 |
| C_29_H_40_NO_19_ | 706.2204 | 1.3181612 | 2.00E-19 | -15.8085 |
| C_29_H_40_NO_19_ | 706.2204 | 4.260001 | 1.57E-17 | -14.2101 |
| N.a. | 706.2204 | 1.41 | 7.50E-20 | -14.6672 |
| C_29_H_25_N_17_O_6_ | 707.2176 | 0.9729997 | 9.38E-08 | 6.532881 |
| N.a. | 707.2233 | 3.5739996 | 7.50E-20 | -14.6672 |
| C_22_H_29_N_17_O_11_ | 707.2237 | 1.3419994 | 7.91E-15 | -13.3793 |
| C_27_H_23_N_20_O_5_ | 707.2259 | 1.34 | 8.29E-14 | -13.2047 |
| C_38_H_70_C_l3_N_2_OS | 707.4233 | 3.8929994 | 8.52E-20 | -15.8073 |
| C_39_H_35_ClN_4_O_4_S_2_ | 722.177 | 0.9191032 | 0.003385 | -5.50532 |
| C_32_H_30_N_6_O_14_ | 722.1814 | 1.2847505 | 1.75E-08 | -14.5109 |
| C_31_H_34_N_2_O_18_ | 722.1823 | 1.3037498 | 0.002779 | -6.91759 |
| C_23_H_38_NO_26_ | 744.1671 | 0.9050001 | 6.42E-25 | -27.281 |
| N.a. | 801.7323 | 4.118838 | 1.46E-10 | -16.7014 |
| N.a. | 886.4582 | 4.3844447 | 5.66E-09 | -15.5799 |
| N.a. | 897.5483 | 4.25 | 4.54E-15 | -16.8759 |
| N.a. | 922.6051 | 4.9980006 | 5.63E-12 | -12.6922 |
| N.a. | 961.669 | 4.122001 | 2.18E-27 | -29.4585 |
| N.a. | 961.7558 | 4.1129994 | 7.50E-20 | -14.6672 |
| N.a. | 961.7827 | 4.108001 | 7.50E-20 | -14.6672 |
| N.a. | 961.8144 | 4.1169987 | 7.50E-20 | -14.6672 |
| N.a. | 990.6642 | 7.229003 | 2.08E-13 | -13.1813 |
| N.a. | 1014.5623 | 3.9790018 | 1.50E-25 | -27.7405 |
| N.a. | 1064.2913 | 1.0345 | 6.06E-11 | -17.3128 |
| N.a. | 1064.2924 | 1.0229994 | 1.43E-07 | -14.9095 |
| N.a. | 1064.2971 | 1.1369997 | 0.043665 | -4.10097 |
| N.a. | 1064.2979 | 1.2523218 | 4.14E-11 | -18.1533 |
| N.a. | 1064.2996 | 1.3309999 | 1.39E-07 | -10.5067 |
| **Continued** |  |  |  |  |
| **Compound** | **Monoisotopic Mass (Da)** | **Retention Time (min)** | **p (Corr)** | **LnFC**  **DD vs. ZG** |
| N.a. | 1064.3007 | 1.2411997 | 2.67E-23 | -28.0852 |
| N.a. | 1113.6392 | 4.039001 | 3.19E-16 | -18.7392 |
| N.a. | 1162.6167 | 3.5640008 | 3.00E-22 | -16.251 |
| N.a. | 1226.7231 | 4.1448584 | 4.42E-11 | -16.4664 |
| N.a. | 1259.8013 | 4.111 | 2.67E-05 | -7.93195 |
| N.a. | 1270.2173 | 3.6289995 | 0.011233 | 2.849572 |
| N.a. | 1275.8022 | 3.7140186 | 2.11E-09 | -14.4403 |
| N.a. | 1289.8173 | 3.8284416 | 4.78E-08 | -14.3357 |
| N.a. | 1313.7579 | 4.1702595 | 6.95E-11 | -17.7549 |
| N.a. | 1362.7352 | 3.736 | 1.33E-10 | -14.1468 |
| N.a. | 1406.4097 | 1.0230005 | 1.09E-08 | -16.3222 |
| N.a. | 1406.4124 | 1.1510005 | 0.017569 | 3.121377 |
| N.a. | 1406.4124 | 1.1559998 | 0.017965 | 3.108504 |
| N.a. | 1406.4124 | 1.1994258 | 1.20E-11 | -15.1806 |
| N.a. | 1406.4126 | 1.087 | 0.009247 | -5.52003 |
| N.a. | 1406.4137 | 1.2414516 | 2.65E-13 | -20.3235 |
| N.a. | 1406.415 | 1.271 | 1.13E-08 | -11.1338 |
| N.a. | 1406.4181 | 1.3209995 | 8.95E-11 | -12.1847 |
| N.a. | 1406.4214 | 1.384 | 1.75E-16 | -13.8733 |
| N.a. | 1692.9023 | 3.595209 | 3.31E-14 | -18.6042 |
| N.a. | 1748.7087 | 4.0400004 | 4.27E-22 | -15.1653 |
| N.a. | 1950.58 | 4.301001 | 1.63E-10 | -11.5877 |
| N.a. | 1990.5887 | 4.359 | 0.021795 | 2.557115 |
| N.a. | 1991.088 | 4.3570004 | 0.005678 | 3.117877 |
| N.a. | 2020.039 | 4.071999 | 9.67E-08 | -10.1643 |
| N.a. | 2123.134 | 4.0770016 | 1.09E-17 | -19.7956 |
| N.a. | 2123.182 | 4.2606435 | 1.32E-11 | -17.7457 |
| N.a. | 2123.688 | 4.262275 | 1.10E-09 | -12.9231 |
| N.a. | 2148.3435 | 4.931 | 7.02E-16 | -16.2583 |
| N.a. | 2148.711 | 4.9355373 | 7.08E-19 | -16.0366 |
| N.a. | 2193.2449 | 3.8239992 | 1.34E-18 | -16.2151 |
| N.a. | 2210.2715 | 3.703327 | 3.43E-11 | -16.2195 |
| N.a. | 2236.2126 | 4.120902 | 7.38E-18 | -15.4046 |
| N.a. | 2290.2952 | 3.6019993 | 1.27E-09 | -11.3242 |
| N.a. | 2363.7065 | 4.060999 | 1.12E-06 | -9.26624 |
| N.a. | 2364.191 | 4.0741434 | 1.96E-21 | -17.9356 |
| N.a. | 2364.6958 | 4.0705104 | 6.55E-12 | -13.9898 |
| N.a. | 2366.3738 | 3.6240003 | 1.61E-15 | -13.7246 |
| N.a. | 2378.371 | 3.8235366 | 5.49E-10 | -15.6534 |
| N.a. | 2379.3708 | 3.8290007 | 1.96E-21 | -24.8417 |
| N.a. | 2404.6958 | 4.1126065 | 2.01E-07 | -8.43964 |
| N.a. | 2404.6995 | 4.1050014 | 0.021631 | 2.57356 |
| N.a. | 2451.2576 | 4.2070003 | 3.82E-25 | -15.7042 |
| N.a. | 2479.3347 | 4.074999 | 1.92E-15 | -16.7411 |
| N.a. | 2540.4355 | 3.6357422 | 1.63E-15 | -13.6026 |
| **Continued** |  |  |  |  |
| **Compound** | **Monoisotopic Mass (Da)** | **Retention Time (min)** | **p (Corr)** | **LnFC**  **DD vs. ZG** |
|  |  |  |  |  |
| N.a. | 2541.4373 | 3.6279988 | 0.002796 | 3.391507 |
| N.a. | 2554.453 | 3.7372277 | 4.72E-07 | -14.7344 |
| N.a. | 2589.1409 | 3.7358265 | 1.08E-11 | -17.7526 |
| N.a. | 2723.312 | 3.8685672 | 6.59E-12 | -16.9307 |
| N.a. | 2846.5325 | 4.5589986 | 6.94E-18 | -16.6784 |
| N.a. | 2864.5427 | 3.693508 | 1.46E-10 | -15.3104 |
| N.a. | 2865.5447 | 3.685999 | 0.015099 | -4.42162 |
| N.a. | 2906.3364 | 3.82 | 1.14E-23 | -22.1112 |
| N.a. | 2907.3467 | 3.806 | 1.94E-06 | -9.11822 |
| N.a. | 2914.8474 | 4.042 | 1.90E-38 | -16.6641 |
| N.a. | 3030.078 | 4.0869985 | 7.48E-27 | -19.4738 |
| N.a. | 3137.587 | 4.2769995 | 3.24E-12 | -12.7198 |
| N.a. | 3191.5728 | 4.143999 | 7.50E-20 | -14.6672 |
| N.a. | 3222.055 | 4.933999 | 1.73E-15 | -20.2794 |
| N.a. | 3222.5354 | 4.9270015 | 1.69E-06 | -9.73701 |
| N.a. | 3223.0522 | 4.9566855 | 6.21E-10 | -11.6672 |
| N.a. | 3229.1187 | 5.004937 | 1.78E-08 | -13.5015 |
| N.a. | 3229.62 | 5.0070004 | 1.59E-09 | -15.8745 |
| N.a. | 3352.867 | 4.384317 | 7.52E-15 | -19.66 |
| N.a. | 3353.0662 | 4.378999 | 1.13E-12 | -12.7903 |
| N.a. | 3384.614 | 4.9600005 | 4.27E-22 | -15.2615 |
| N.a. | 3481.912 | 4.3830013 | 1.91E-21 | -17.9427 |
| N.a. | 3599.649 | 3.9369986 | 4.45E-29 | -16.279 |
| N.a. | 3901.1438 | 4.298998 | 1.22E-17 | -14.2211 |
| N.a. | 3980.171 | 4.358001 | 3.41E-05 | 4.822392 |
| N.a. | 3981.1716 | 4.3570004 | 7.46E-05 | 4.573617 |
| N.a. | 3996.1929 | 4.2949986 | 5.66E-09 | -11.0985 |
| N.a. | 3997.1797 | 4.3092327 | 7.73E-15 | -17.5588 |
| N.a. | 4012.1926 | 4.2310004 | 8.09E-06 | -8.65443 |
| N.a. | 4013.1943 | 4.2425985 | 5.23E-12 | -18.5911 |
| N.a. | 4084.3936 | 4.3150005 | 7.50E-20 | -14.6672 |
| N.a. | 4117.2544 | 4.313782 | 2.18E-27 | -29.3591 |
| N.a. | 4118.228 | 4.308218 | 1.67E-10 | -13.4062 |
| N.a. | 4124.8525 | 4.3378887 | 1.21E-16 | -20.1513 |
| N.a. | 4230.0957 | 4.167158 | 1.42E-14 | -16.1153 |
| N.a. | 4246.36 | 4.2631135 | 2.87E-10 | -16.1314 |
| N.a. | 4269.7993 | 4.543999 | 7.52E-16 | -16.2685 |
| N.a. | 4305.2656 | 4.4252496 | 1.29E-11 | -18.6856 |
| N.a. | 4371.7695 | 4.041 | 1.13E-25 | -15.702 |
| N.a. | 4482.4785 | 4.4700007 | 6.49E-17 | -17.3142 |
| N.a. | 4680.246 | 4.1746807 | 1.46E-12 | -18.8088 |
| N.a. | 4728.3613 | 4.0708985 | 2.13E-19 | -17.3089 |
| N.a. | 4807.34 | 4.1050014 | 0.002521 | 3.432525 |
| **Continued** |  |  |  |  |

| Compound | Monoisotopic Mass (Da) | Retention Time (min) | p (Corr) | LnFC  July vs. June | LnFC  August vs. June |
| --- | --- | --- | --- | --- | --- |

|  |  |  |  |  |
| --- | --- | --- | --- | --- |
| N.a. | 4808.377 | 4.1123357 | 0.006088 | -5.15019 |
| N.a. | 5132.453 | 4.166 | 4.32E-19 | -22.1184 |
| N.a. | 5133.4404 | 4.153 | 4.41E-08 | -10.3337 |

Milk Composition Database (MCDB) and Bovine Metabolome Database (BMDB) were used for the identification. n.a. = compounds for which it was not possible to assign a name and/or a molecular formula. p(Corr) = corrected p-value obtained after application of Benjamini-Hochberg procedure. LnFC = Natural logarithm of Fold Change.

**TableS4.** Metabolites obtained from LC-MS data (positive mode), that are differentially accumulated in milk samples from buffaloes of DD Group (fed a total mixed ratio (TMR)) for the whole experimental period (June, July and August). Identifications were performed by comparing results with known compounds present in freely available electronic databases.

| Compound | Monoisotopic Mass (Da) | Retention Time (min) | p (Corr) | LnFC  July vs. June | LnFC  August vs. June |
| --- | --- | --- | --- | --- | --- |
| δ-Valerobetaine | 159.1252 | 1.0007333 | 1.80E-07 | 7.1799765 | -3.2817602 |
| L-Carnitine | 161.1045 | 1.0559787 | 1.94E-07 | 7.0949345 | -3.4304905 |
| C_6_NO_3_S_2_ | 197.9329 | 0.839711 | 7.44E-08 | 7.3085117 | -3.4423146 |
| N.a. | 203.1157 | 1.0155555 | 6.43E-08 | 8.351495 | -2.4789858 |
| Acetylcarnitine | 203.1163 | 1.1773789 | 2.90E-24 | -14.375848 | -25.062004 |
| N.a. | 203.1165 | 1.6930006 | 7.44E-08 | 7.5317 | -3.1544552 |
| Propionylcarnitine | 217.1312 | 1.2245564 | 5.62E-09 | 9.049169 | -3.0859761 |
| C_11_H_15_N_5_ | 217.1325 | 1.3125 | 9.77E-10 | -12.786933 | -3.4296365 |
| N.a. | 231.1484 | 1.8979995 | 7.44E-08 | 7.5317 | -3.1544552 |
| Butyrylcarnitine | 231.1485 | 1.5028224 | 6.27E-08 | 6.968273 | -3.7872849 |
| 2-Methylbutyrylcarnitine | 245.1644 | 3.6959107 | 2.57E-09 | 8.384206 | -3.6744347 |
| C_13_H_19_N_5_ | 245.1646 | 3.7449772 | 3.78E-08 | 7.54813 | -3.5526676 |
| Glycerophosphocholine | 257.1016 | 1.1453104 | 1.02E-20 | 28.734497 | 10.195721 |
| N.a. | 257.1023 | 0.89100015 | 7.37E-30 | 26.763056 | -3.1544552 |
| C_16_H_35_NO | 257.2735 | 5.841002 | 3.53E-21 | -11.865864 | -22.55202 |
| C_16_H_17_N_5_ | 279.1485 | 3.9568083 | 1.15E-06 | 7.487604 | -2.832951 |
| C_16_H_33_N_4_ | 281.2724 | 11.115982 | 1.49E-05 | 1.008894 | -10.354231 |
| C_12_H_11_N_2_O_7_ | 295.0573 | 0.88715506 | 8.33E-08 | 7.482628 | -3.3106055 |
| C_19_H_21_NO_4_ | 327.1469 | 1.4449993 | 1.74E-03 | 0.49136972 | -8.953049 |
| N.a. | 337.3333 | 11.302001 | 7.44E-08 | 7.5317 | -3.1544552 |
| C_22_H_43_NO | 337.3334 | 12.103245 | 1.82E-14 | -2.1301994 | -19.10738 |
| C_20_H_41_N_4_ | 337.3334 | 10.832 | 1.40E-14 | 7.5317 | 16.256222 |
| N.a. | 337.3335 | 10.832 | 1.40E-14 | 7.5317 | 16.256222 |
| N.a. | 337.3335 | 10.832 | 1.40E-14 | 7.5317 | 16.256222 |
| N.a. | 337.3336 | 10.839997 | 2.48E-11 | 7.5317 | 15.162354 |
| N.a. | 337.3337 | 10.609997 | 7.44E-08 | 7.5317 | -3.1544552 |
| N.a. | 337.334 | 10.832 | 1.50E-14 | 7.5317 | 16.229439 |
| C_15_H_17_N_6_OS_2_ | 361.0904 | 0.90459335 | 5.31E-08 | 7.5317 | 10.936752 |
| C_11_H_17_N_6_O_6_S | 361.0909 | 0.9945335 | 3.02E-06 | 6.0738955 | -4.868689 |
| C_10_H_20_ClN_3_O_9_ | 361.0937 | 1.0332954 | 1.55E-05 | -9.761623 | -2.6047764 |
| N.a. | 364.0967 | 13.493669 | 1.69E-12 | 6.6470633 | -13.318625 |
| N.a. | 364.0979 | 1.198756 | 2.90E-24 | -14.315845 | -25.663609 |
| C_9_H_20_N_2_O_13_ | 364.0994 | 0.9367778 | 5.60E-08 | 9.101851 | -1.2736893 |
| C_14_H_20_O_11_ | 364.1003 | 4.218001 | 1.22E-05 | -0.577855 | -11.26401 |
| N.a. | 364.1007 | 3.908999 | 2.50E-30 | 27.8933 | -3.1544552 |
| N.a. | 364.1014 | 1.4799997 | 7.44E-08 | 7.5317 | -3.1544552 |
| C_18_H_9_ N_10_ | 365.1026 | 0.95999956 | 2.54E-16 | 7.5317 | 17.741135 |
| N.a. | 365.1028 | 1.1089998 | 5.20E-07 | 7.5317 | -2.5718384 |
| C_16_H_19_N_3_O_5_S | 365.1038 | 1.2593789 | 7.44E-08 | 7.5317 | -3.1544552 |
| N.a. | 365.104 | 1.3000001 | 7.44E-08 | 7.5317 | -3.1544552 |
| C_12_H_16_N_2_O_12_ | 380.0714 | 0.9055778 | 1.28E-09 | 8.667333 | -4.1420536 |
| C_17_H_16_O_10_ | 380.0743 | 3.9820485 | 1.56E-07 | 2.521906 | -13.725642 |
| N.a. | 380.0749 | 1.472 | 7.44E-08 | 7.5317 | -3.1544552 |
| N.a. | 380.075 | 1.3670002 | 7.44E-08 | 7.5317 | -3.1544552 |
| N.a. | 382.0713 | 0.97100025 | 4.76E-05 | 7.5317 | -0.7621622 |
| C_11_H_16_N_3_O_12_ | 382.0719 | 0.9070005 | 6.27E-08 | 7.7927513 | -3.3169274 |
| N.a. | 382.0751 | 1.2700003 | 7.44E-08 | 7.5317 | -3.1544552 |
| N.a. | 441.871 | 0.84218407 | 6.14E-06 | 5.8509 | -4.10118 |
| Continued |  |  |  |  |  |
| Compound | **Monoisotopic Mass (Da)** | **Retention Time (min)** | **p (Corr)** | **LnFC**  **July vs. June** | **LnFC**  **August vs. June** |
| C_26_H_43_O_7_ | 467.3011 | 6.2700005 | 1.79E-13 | 7.5317 | 15.186086 |
| C_15_H_33_N_17_O | 467.3013 | 6.261647 | 1.69E-08 | 9.402738 | -1.0270424 |
| N.a. | 477.2848 | 6.7319984 | 1.79E-13 | 7.5317 | 15.346165 |
| C_27_H_4_O_7_ | 477.2854 | 6.71787 | 1.26E-02 | 6.3651 | 0.29365635 |
| C_27_H_43_O_7_ | 479.3007 | 7.423227 | 5.80E-08 | 8.329376 | -2.9431577 |
| C_23_H_53_N_4_O_6_ | 481.3991 | 10.496558 | 6.18E-29 | 7.2588205 | -21.98943 |
| N.a. | 495.332 | 7.472 | 7.44E-08 | 7.5317 | -3.1544552 |
| C_24_H_50_NO_7_P | 495.3321 | 7.4190025 | 7.44E-08 | 7.5317 | -3.1544552 |
| C_15_H_35_N_20_ | 495.3361 | 7.0122313 | 1.40E-08 | 8.900309 | -3.6001844 |
| C_16_H_41_N_13_O_5_ | 495.3377 | 7.2059755 | 3.46E-08 | 7.3672247 | -3.6795177 |
| C_15_H_43_N_12_O_8_ | 519.3321 | 6.800998 | 5.36E-13 | 7.5317 | 14.866067 |
| N.a. | 521.3476 | 7.7940025 | 7.44E-08 | 7.5317 | -3.1544552 |
| C_26_H_52_NO_7_P | 521.3478 | 7.7590017 | 7.44E-08 | 7.5317 | -3.1544552 |
| C_17_H_37_N_20_ | 521.348 | 7.3493524 | 1.94E-02 | 0.7726867 | -5.465622 |
| C_19_H_39_N_17_O | 521.3522 | 7.541623 | 7.44E-08 | 7.376052 | -3.1528516 |
| C_23_H_55_N_7_O_6_ | 525.4268 | 10.425175 | 6.82E-29 | 7.3650017 | -21.81153 |
| N.a. | 706.2145 | 1.0478964 | 6.45E-20 | 9.272899 | -21.648865 |
| C_21_H_42_N_2_O_24_ | 706.215 | 0.9727777 | 2.40E-08 | 8.692579 | -2.0754938 |
| N.a. | 706.215 | 1.2373104 | 1.87E-13 | -10.351169 | -21.6488 |
| C_27_H_38_N_4_O_18_ | 706.2199 | 3.9531353 | 6.29E-08 | 8.389143 | -2.9542718 |
| N.a. | 706.2199 | 3.928001 | 2.93E-15 | 7.5317 | 17.006239 |
| C_29_H_40_NO_19_ | 706.2202 | 3.928001 | 1.98E-15 | 7.5317 | 17.074657 |
| N.a. | 706.2204 | 4.25 | 3.95E-05 | 0.94127214 | -9.287798 |
| N.a. | 706.2204 | 1.41 | 7.44E-08 | 7.5317 | -3.1544552 |
| C_29_H_25_N_17_O_6_ | 707.2176 | 0.97075826 | 4.26E-27 | 28.421173 | 18.045654 |
| C_29_H_32_N_5_O_17_ | 722.1816 | 1.1828568 | 1.49E-10 | 15.248533 | -3.7493668 |
| C_26_H_24_N_15_O_11_ | 722.1819 | 0.9461895 | 4.38E-12 | 8.974225 | -8.478073 |
| N.a. | 823.8199 | 4.324999 | 7.44E-08 | 7.5317 | -3.1544552 |
| N.a. | 823.932 | 4.327001 | 7.44E-08 | 7.5317 | -3.1544552 |
| N.a. | 849.3858 | 4.2647567 | 7.44E-08 | 7.5317 | -3.1544552 |
| N.a. | 849.4098 | 4.277414 | 7.44E-08 | 7.5317 | -3.1544552 |
| N.a. | 920.8113 | 4.9550014 | 7.44E-08 | 7.5317 | -3.1544552 |
| N.a. | 945.824 | 4.093001 | 7.44E-08 | 7.5317 | -3.1544552 |
| N.a. | 961.67 | 4.1298947 | 7.37E-30 | 7.7201157 | -21.223635 |
| N.a. | 961.7558 | 4.1129994 | 7.44E-08 | 7.5317 | -3.1544552 |
| N.a. | 961.7827 | 4.108001 | 7.44E-08 | 7.5317 | -3.1544552 |
| N.a. | 961.8144 | 4.1169987 | 7.44E-08 | 7.5317 | -3.1544552 |
| N.a. | 961.9643 | 4.1367607 | 7.44E-08 | 7.5317 | -3.1544552 |
| N.a. | 990.524 | 4.078001 | 3.18E-10 | 9.905515 | -3.1544552 |
| N.a. | 1014.5603 | 3.9887564 | 1.03E-13 | 7.5933366 | -12.209853 |
| N.a. | 1048.3138 | 1.0452907 | 1.84E-07 | -3.8873472 | -13.657738 |
| N.a. | 1064.294 | 1.0533289 | 8.87E-08 | 1.6877232 | -11.728153 |
| N.a. | 1064.2971 | 1.1621524 | 1.04E-06 | 13.321977 | 3.0587642 |
| N.a. | 1113.6295 | 4.057457 | 8.50E-11 | 5.9171534 | -9.747455 |
| N.a. | 1226.7174 | 4.1503677 | 3.18E-12 | 9.913719 | -4.791151 |
| N.a. | 1270.2173 | 3.6422298 | 5.56E-05 | 9.696919 | 8.196169 |
| N.a. | 1275.8007 | 3.7225218 | 6.37E-06 | 8.734513 | -5.169435 |
| N.a. | 1289.8167 | 3.8404384 | 0.003392245 | 5.1447763 | -4.8319597 |
| N.a. | 1406.408 | 1.1151106 | 1.61E-08 | -11.103149 | -3.348785 |
| N.a. | 1406.4094 | 1.0752586 | 5.84E-16 | 7.800009 | -15.3697405 |
| N.a. | 1406.4113 | 1.1458108 | 1.69E-12 | 19.189314 | 6.279318 |
| Continued |  |  |  |  |  |
| Compound | **Monoisotopic Mass (Da)** | **Retention Time (min)** | **p (Corr)** | **LnFC**  **July vs. June** | **LnFC**  **August vs. June** |
| N.a. | 1990.5887 | 4.359 | 2.46E-12 | 7.5317 | 14.069889 |
| N.a. | 1991.088 | 4.370896 | 1.90E-10 | 5.2875686 | 12.3865185 |
| N.a. | 2123.688 | 4.2676377 | 9.31E-13 | 8.277486 | -5.392935 |
| N.a. | 2148.0083 | 4.960001 | 7.44E-08 | 7.5317 | -3.1544552 |
| N.a. | 2153.315 | 5.0120006 | 7.44E-08 | 7.5317 | -3.1544552 |
| N.a. | 2210.2715 | 3.7106907 | 4.98E-10 | 9.192723 | -7.9438725 |
| N.a. | 2236.2153 | 4.129192 | 1.81E-11 | 8.187832 | -4.3438826 |
| N.a. | 2237.227 | 4.113999 | 6.57E-10 | 7.5317 | 12.584106 |
| N.a. | 2378.3684 | 3.8307936 | 3.56E-09 | 8.760979 | -5.4073944 |
| N.a. | 2404.6958 | 4.1050014 | 9.37E-13 | 7.5317 | 14.092356 |
| N.a. | 2404.6995 | 4.1050014 | 9.38E-13 | 7.5317 | 14.086334 |
| N.a. | 2451.2595 | 4.2155695 | 7.17E-18 | 7.87351 | -11.161143 |
| N.a. | 2479.3398 | 4.0910015 | 2.74E-06 | 5.4789176 | -5.2072377 |
| N.a. | 2509.1785 | 3.6600018 | 1.34E-07 | 6.4686184 | -4.217537 |
| N.a. | 2540.4353 | 3.643178 | 1.02E-06 | 6.097842 | -4.219466 |
| N.a. | 2541.4373 | 3.640501 | 3.34E-07 | -9.793123 | -2.4205437 |
| N.a. | 2554.453 | 3.7448423 | 4.68E-02 | 3.6014874 | -3.75454 |
| N.a. | 2589.1436 | 3.744825 | 5.30E-09 | 8.514168 | -5.721839 |
| N.a. | 2796.24 | 3.597 | 7.03E-10 | 8.759455 | -3.1544552 |
| N.a. | 2864.5427 | 3.7006342 | 7.44E-08 | 6.7201552 | -7.842429 |
| N.a. | 2865.5447 | 3.685999 | 2.07E-04 | 7.5317 | 7.091153 |
| N.a. | 2906.3418 | 3.8226318 | 9.38E-16 | 6.532012 | -15.141687 |
| N.a. | 2980.3606 | 3.7169986 | 1.44E-16 | 8.4439335 | -11.25705 |
| N.a. | 3137.587 | 4.2914667 | 2.37E-06 | 7.92904 | -3.332292 |
| N.a. | 3222.0188 | 4.939001 | 6.69E-19 | 20.035137 | -3.1544552 |
| N.a. | 3222.5354 | 4.9270015 | 2.40E-03 | 7.5317 | 1.7757592 |
| N.a. | 3229.1187 | 5.0028276 | 4.92E-11 | 18.597057 | 3.6849031 |
| N.a. | 3229.62 | 5.00393 | 4.36E-11 | 18.566786 | 3.6079602 |
| N.a. | 3385.1165 | 4.977 | 7.44E-08 | 7.5317 | -3.1544552 |
| N.a. | 3481.9194 | 4.393999 | 1.77E-07 | 3.2817316 | -7.4044237 |
| N.a. | 3852.0886 | 4.4660854 | 1.69E-08 | 7.7759514 | -6.689734 |
| N.a. | 3924.9683 | 4.4100013 | 3.52E-08 | 8.760092 | -3.1544552 |
| N.a. | 3980.171 | 4.370087 | 2.36E-07 | 9.651923 | -1.9612556 |
| N.a. | 3981.1765 | 4.3674994 | 3.70E-11 | -12.91152 | -4.356831 |
| N.a. | 3996.212 | 4.3067236 | 8.16E-10 | 14.295066 | 0.41427422 |
| N.a. | 4245.353 | 4.2684526 | 3.25E-16 | 7.730958 | -10.30245 |
| N.a. | 4247.3545 | 4.2790003 | 8.09E-22 | -10.258647 | -20.944801 |
| N.a. | 4304.276 | 4.435999 | 9.20E-18 | 19.102806 | -3.1544552 |
| N.a. | 4309.0254 | 4.231547 | 7.27E-16 | 7.6743603 | -7.5402274 |
| N.a. | 4570.4663 | 4.3213267 | 7.20E-12 | 5.3849044 | -8.966899 |
| N.a. | 4679.4775 | 4.191001 | 1.53E-18 | -7.532049 | -18.218204 |
| N.a. | 4728.371 | 4.077 | 4.55E-10 | 11.606588 | -3.0320702 |
| N.a. | 4807.3345 | 4.1207566 | 5.89E-10 | 7.124547 | -4.53226 |
| N.a. | 4808.377 | 4.1154103 | 3.93E-15 | 18.060379 | 14.689636 |
| N.a. | 6298.9873 | 4.132691 | 3.45E-08 | 7.0490174 | -9.713106 |
| N.a. | 6446.003 | 4.942001 | 7.44E-08 | 7.5317 | -3.1544552 |
|  |  |  |  |  |  |

Milk Composition Database (MCDB) and Bovine Metabolome Database (BMDB) were used for the identification. n.a. = compounds for which it was not possible to assign a name and/or a molecular formula. p(Corr) = corrected p-value obtained after application of Benjamini-Hochberg procedure. LnFC = Natural logarithm of Fold Change.

**TableS5.** Metabolites obtained from LC-MS data (positive mode), that are differentially accumulated in milk samples from buffaloes of ZG Group (fed TMR+30% of green forage) for the whole experimental period (June, July and August). Identifications were performed by comparing results with known compounds present in freely available electronic databases.

| Compound | Monoisotopic Mass (Da) | | Retention Time (min) | | | p (Corr) | | LnFC  July vs. June | | LnFC  August vs. June | |
| --- | --- | --- | --- | --- | --- | --- | --- | --- | --- | --- | --- |
| C_7_H_13_NO_2_ | 143.094 | | 1.1298665 | | | 7.61E-20 | | -6.0399 | | -16.543491 | |
| γ-Butyrobetaine | 145.1099 | | 1.0868727 | | | 0.004312 | | 3.0676727 | | 3.0186539 | |
| C_11_H_15_N_5_ | 217.1326 | | 1.3246461 | | | 0.027089 | | 1.5627022 | | 2.498726 | |
| Propionylcarnitine | 217.1326 | | 1.1965 | | | 3.56E-28 | | 1.3566017 | | -16.55081 | |
| Butyrylcarnitine | 231.1482 | | 1.521 | | | 0 | | 23.485516 | | 1.4046552 | |
| Glycerophosphocholine | 257.1031 | | 1.2539996 | | | 2.01E-23 | | 10.945495 | | -10.318822 | |
| C_16_H_35_NO | 257.2736 | | 5.8688354 | | | 1.44E-20 | | -18.408669 | | -15.294502 | |
| C_22_H_43_NO | 337.3332 | | 12.114232 | | | 1.05E-05 | | -8.801245 | | -9.754713 | |
| N.a. | 337.3345 | | 10.812001 | | | 9.77E-20 | | 15.727373 | | 1.4046552 | |
| C_10_H_21_Cl_2_N_5_O_5_ | 361.0907 | | 0.9232858 | | | 6.01E-29 | | 3.2408314 | | -13.795168 | |
| C_9_H_20_N_2_O_13_ | 364.0968 | | 13.476519 | | | 0 | | 22.134388 | | 21.910963 | |
| C_9_H_20_N_2_O_13_ | 364.0991 | | 0.9700963 | | | 0 | | 25.239868 | | 25.05586 | |
| N.a. | 364.1008 | | 1.2755159 | | | 0.025733 | | 1.4046552 | | 3.18532 | |
| C_17_H_13_N_6_O_4_ | 365.1023 | | 1.1176244 | | | 8.24E-20 | | 12.5119095 | | -8.423193 | |
| C_13_H_12_N_6_O_8_ | 380.072 | | 0.90399957 | | | 0 | | 25.775164 | | 25.41212 | |
| C_11_H_16_N_3_O_12_ | 382.0727 | | 0.9060001 | | | 0 | | 1.4046552 | | 21.996456 | |
| C_15_H_33_N_17_O | 467.3041 | | 6.2531323 | | | 0 | | 19.672428 | | 19.869062 | |
| N.a. | 477.2885 | | 6.7082047 | | | 1.94E-04 | | -4.4069996 | | 2.0699577 | |
| N.a. | 477.289 | | 6.695603 | | | 2.52E-26 | | -14.528357 | | -16.211752 | |
| C_14_H_29_N_20_ | 477.2891 | | 6.6991916 | | | 3.34E-22 | | -2.6662025 | | -16.82933 | |
| C_14_H_31_N_20_ | 479.3001 | | 7.3203816 | | | 3.99E-20 | | 2.0547836 | | 17.486204 | |
| C_15_H_35_N_20_ | 495.3355 | | 7.014931 | | | 7.97E-15 | | 15.253804 | | 13.579543 | |
| C_17_H_37_N_17_O | 495.3358 | | 7.1981626 | | | 0 | | 22.800106 | | 22.942797 | |
| C_19_H_37_N_17_O | 519.3336 | | 6.783684 | | | 1.54E-05 | | -3.1968193 | | 6.186948 | |
| C_26_H_52_NO_7_P | 521.3469 | | 7.3831215 | | | 0.005144 | | 3.4975975 | | 5.789707 | |
| C_17_H_37_N_20_ | 521.3528 | | 7.5356765 | | | 0 | | 21.138664 | | 21.935192 | |
| C_23_H_19_N_19_OS | 609.1764 | | 13.708005 | | | 4.21E-09 | | 10.983728 | | 1.4046552 | |
| C_24_H_33_N_9_O_16_ | 703.2057 | | 1.1193908 | | | 1.16E-11 | | 12.015655 | | 1.4046552 | |
| C_21_H_42_N_2_O_24_ | 706.2171 | | 0.97900003 | | | 0 | | 24.706505 | | 24.842999 | |
| C_27_H_38_N_4_O_18_ | 706.2192 | | 3.9841952 | | | 0.036691 | | 1.5426121 | | 2.465765 | |
| C_21_H_4_ N_2_O_24_ | 707.221 | | 0.98200023 | | | 0 | | 22.542776 | | 1.4046552 | |
| C_24_H_22_N_18_O_10_ | 722.1799 | | 1.128267 | | | 3.87E-20 | | 18.67867 | | 10.379324 | |
| C_29_H_32_N_5_O_17_ | 722.1818 | | 1.2507637 | | | 8.19E-05 | | 7.5167513 | | 9.881944 | |
| C_29_H_22_N_16_O_8_ | 722.1854 | | 0.90399975 | | | 2.73E-39 | | 16.939804 | | 1.4046552 | |
| C_2_ H_32_N_8_O_16_S | 744.1664 | | 0.9117366 | | | 1.26E-23 | | -13.8803835 | | -1.2665939 | |
| N.a. | 897.543 | | 4.2409344 | | | 0.034494 | | 2.540569 | | 3.6133268 | |
| N.a. | 961.6714 | | 4.1231203 | | | 1.18E-29 | | 19.599703 | | 16.195902 | |
| N.a. | 1064.296 | | 1.1747036 | | | 3.55E-11 | | 13.250895 | | 8.768625 | |
| N.a. | 1065.297 | | 1.0934683 | | | 4.59E-08 | | 9.005949 | | 1.4046552 | |
| N.a. | 1270.222 | | 3.6818972 | | | 0.005469 | | -0.27811003 | | -5.1747055 | |
| N.a. | 1406.406 | | 1.0200003 | | | 2.39E-07 | | 1.4046552 | | 8.543841 | |
| N.a. | 1406.41 | | 1.1972622 | | | 3.68E-21 | | 17.03016 | | 18.207544 | |
| N.a. | 1406.412 | | 1.1578646 | | | 2.06E-09 | | 10.830166 | | 8.971857 | |
| N.a. | 1990.594 | | 4.3739996 | | | 0.013899 | | 3.214796 | | 1.4046552 | |
| N.a. | 2123.132 | | 4.0817204 | | | 3.93E-06 | | 9.768696 | | 6.533061 | |
| N.a. | 2148.349 | | 4.9704285 | | | 0.029882 | | -4.7513056 | | -3.1602428 | |
| N.a. | 2236.216 | | 4.1275167 | | | 0 | | 20.65015 | | 19.71069 | |
| Continued | |  | |  |  | |  | |  | |  |
| Compound | | **Monoisotopic Mass (Da)** | | **Retention Time (min)** | **p (Corr)** | | **LnFC**  **July vs. June** | | **LnFC**  **August vs. June** | |  |
|  |  | |  | | |  | |  | |  | |
| N.a. | 2378.368 | | 3.8364575 | | | 2.12E-11 | | 13.797716 | | 12.611744 | |
| N.a. | 2479.335 | | 4.074999 | | | 0.010653 | | 1.4046552 | | 3.4784908 | |
| N.a. | 2540.436 | | 3.6465805 | | | 3.13E-26 | | 18.381786 | | 18.874205 | |
| N.a. | 2579.358 | | 4.103133 | | | 0.00222 | | -0.5709028 | | -5.576359 | |
| N.a. | 2589.141 | | 3.7459815 | | | 2.57E-11 | | 12.958841 | | 12.618185 | |
| N.a. | 2681.538 | | 3.9199986 | | | 0.013578 | | 3.3153806 | | 1.4046552 | |
| N.a. | 2723.316 | | 3.8899987 | | | 0.010368 | | -0.25525475 | | -4.9768887 | |
| N.a. | 2980.354 | | 3.7187617 | | | 3.25E-07 | | -1.2574301 | | -9.758173 | |
| N.a. | 3229.128 | | 5.032418 | | | 0.028598 | | 4.6308923 | | 5.5233808 | |
| N.a. | 3352.867 | | 4.3914757 | | | 1.20E-08 | | 11.305418 | | 10.344398 | |
| N.a. | 3481.912 | | 4.3830013 | | | 0.001358 | | 1.4046552 | | 4.6800795 | |
| N.a. | 3599.649 | | 3.962036 | | | 0.043844 | | 2.8987513 | | 3.0164146 | |
| N.a. | 3838.335 | | 4.6060014 | | | 0.002185 | | 4.371726 | | 1.4046552 | |
| N.a. | 3980.17 | | 4.3700004 | | | 0 | | 21.852676 | | 1.4046552 | |
| N.a. | 4304.263 | | 4.4299994 | | | 2.39E-12 | | 11.541708 | | 1.4046552 | |
| N.a. | 4305.277 | | 4.572542 | | | 1.05E-12 | | 1.4046552 | | 11.417261 | |
| N.a. | 4309.022 | | 4.2340007 | | | 0 | | 20.048908 | | 1.4046552 | |
| N.a. | 4481.466 | | 4.487 | | | 0.002041 | | 4.69168 | | 1.4046552 | |
| N.a. | 4807.341 | | 4.1230006 | | | 0 | | 20.984499 | | 1.4046552 | |

Milk Composition Database (MCDB) and Bovine Metabolome Database (BMDB) were used for the identification. n.a. = compounds for which it was not possible to assign a name and/or a molecular formula. p(Corr) = corrected p-value obtained after application of Benjamini-Hochberg procedure. LnFC = Natural logarithm of Fold Change.

**TableS6.** Metabolites obtained from LC-MS data (positive mode), that are differentially accumulated in ruminal fluid samples from buffaloes of DD Group (fed a total mixed ratio (TMR)) and ZG Group (fed TMR+30% of green forage). Identifications were performed by comparing results with known compounds present in a freely available electronic database.

| Compound | Monoisotopic Mass (Da) | Retention Time (min) | p (Corr) | LnFC  DD vs. ZG |
| --- | --- | --- | --- | --- |
| C_8_H_8_ | 104.0628 | 2.450535 | 0.002215 | -6.43564 |
| C_3_H_4_Cl_2_S | 141.9398 | 1.123615 | 8.67E-04 | 7.727073 |
| N.a. | 163.9214 | 0.916266 | 0.001942 | -6.51875 |
| 3(2-hydroxyphenyl) propanoate | 165.0338 | 1.393556 | 1.18E-21 | -19.4548 |
| Indoleacrylic acid | 187.0635 | 3.510379 | 0.009355 | -4.18757 |
| C_14_H_31_N | 213.2458 | 5.491746 | 0.002241 | 6.539185 |
| C_14_H_31_NO | 229.2409 | 13.23442 | 4.87E-07 | -13.1356 |
| C_16_H_35_NO | 257.2725 | 13.23294 | 0.043251 | -3.31134 |
| Oleamide | 281.2724 | 11.26612 | 0.048498 | -3.20791 |
| C_18_H_12_N_2_O_2_ | 288.0907 | 3.950001 | 2.11E-17 | 15.25271 |
| C_22_H_43_NO | 337.3353 | 12.24614 | 0.042277 | -4.36269 |
| 20-Carboxy-leukotriene B4 | 366.2024 | 5.368213 | 0.007715 | -6.38134 |
| C_11_H_24_O | 390.2783 | 13.6255 | 3.94E-28 | -22.9184 |
| C_19_H_28_N_7_O_3_ | 402.2263 | 9.748875 | 0.011447 | -4.11332 |
| C_18_H_42_N_12_O_4_S | 522.3183 | 12.33441 | 0.008576 | -5.14405 |
| C_34_H_30_N_8_ | 550.2598 | 11.01834 | 0.005862 | -5.75865 |

Bovine Metabolome Database (BMDB) were used for the identification. n.a. = compounds for which it was not possible to assign a name and/or a molecular formula. p(Corr) = corrected p-value obtained after application of Benjamini-Hochberg procedure. LnFC = Natural logarithm of Fold Change.
